# New scoring methodology improves the sensitivity of the Alzheimer’s Disease Assessment Scale-Cognitive subscale (ADAS-Cog) in clinical trials

**DOI:** 10.1186/s13195-015-0151-0

**Published:** 2015-11-12

**Authors:** Nishant Verma, S. Natasha Beretvas, Belen Pascual, Joseph C. Masdeu, Mia K. Markey, The Alzheimer’s Disease Neuroimaging Initiative

**Affiliations:** Department of Biomedical Engineering, The University of Texas at Austin, 107 W. Dean Keeton Street Stop C0800, Austin, TX 78712 USA; NeuroTexas Institute Research Foundation, St. David’s HealthCare, 1015 E. 32nd Street Suite 404, Austin, TX 78705 USA; Department of Educational Psychology, The University of Texas at Austin, 1 University Station D5800, Austin, TX 78712 USA; Nantz National Alzheimer Center, Houston Methodist Neurological Institute, 6560 Fannin Street, Houston, TX 77030 USA; Department of Imaging Physics, The University of Texas MD Anderson Cancer Center, 1400 Pressler Street FCT14.50000, Houston, TX 77030 USA

## Abstract

**Introduction:**

As currently used, the Alzheimer’s Disease Assessment Scale-Cognitive subscale (ADAS-Cog) has low sensitivity for measuring Alzheimer’s disease progression in clinical trials. A major reason behind the low sensitivity is its sub-optimal scoring methodology, which can be improved to obtain better sensitivity.

**Methods:**

Using item response theory, we developed a new scoring methodology (ADAS-CogIRT) for the ADAS-Cog, which addresses several major limitations of the current scoring methodology. The sensitivity of the ADAS-CogIRT methodology was evaluated using clinical trial simulations as well as a negative clinical trial, which had shown an evidence of a treatment effect.

**Results:**

The ADAS-Cog was found to measure impairment in three cognitive domains of memory, language, and praxis. The ADAS-CogIRT methodology required significantly fewer patients and shorter trial durations as compared to the current scoring methodology when both were evaluated in simulated clinical trials. When validated on data from a real clinical trial, the ADAS-CogIRT methodology had higher sensitivity than the current scoring methodology in detecting the treatment effect.

**Conclusions:**

The proposed scoring methodology significantly improves the sensitivity of the ADAS-Cog in measuring progression of cognitive impairment in clinical trials focused in the mild-to-moderate Alzheimer’s disease stage. This provides a boost to the efficiency of clinical trials requiring fewer patients and shorter durations for investigating disease-modifying treatments.

**Electronic supplementary material:**

The online version of this article (doi:10.1186/s13195-015-0151-0) contains supplementary material, which is available to authorized users.

## Introduction

The Alzheimer’s Disease Assessment Scale’s cognitive subscale (ADAS-Cog) is the standard primary cognitive outcome measure for evaluating treatments in clinical trials of mild-to-moderate Alzheimer’s disease. In patients, the ADAS-Cog measures impairment across several cognitive domains that are considered to be affected early and characteristically in Alzheimer’s disease [1]. However, several concerns have been raised recently regarding its sensitivity in measuring progression of cognitive impairment in clinical trials [2–5]. The low sensitivity of the ADAS-Cog has been suggested as a possible reason behind the failure of all clinical trials to date of Alzheimer’s disease treatments [2, 3, 6, 7].

The low sensitivity of the ADAS-Cog is primarily due to most of its items suffering from either floor or ceiling effects in different stages of Alzheimer’s disease [2, 4, 5, 8]. As a result, the ADAS-Cog is limited in measuring progression of cognitive impairment over the course of disease progression. Noting this limitation, research efforts are underway towards modifying the ADAS-Cog and developing new cognitive assessments with better sensitivity [9, 10]. While the importance of developing better assessments cannot be overstated, their in-depth evaluation and eventual utilization in clinical trials is expected to take a significant amount of time. This opens up a parallel research avenue towards improving the application of the ADAS-Cog in clinical trials, which could help make trials more efficient until a better tool is available.

Another major reason behind the low sensitivity of the ADAS-Cog is its suboptimal scoring methodology, which suffers from low accuracy in measuring cognitive impairment. Currently, cognitive impairment is estimated by simply summing scores across the ADAS-Cog items. This methodology suffers from several limitations. Firstly, the current scoring methodology makes an implicit assumption that a single patient trait is measured by the ADAS-Cog. However, psychometric analysis of the ADAS-Cog has suggested that its items measure impairment in multiple cognitive domains [11–13]. The current scoring methodology is equivalent to a weighted summation of impairment in the cognitive domains measured by the ADAS-Cog. In studies of treatments that improve only a subset of cognitive domains, such as improvement in memory but not in language or praxis, the current methodology obscures the detection of treatment effects [14].

Secondly, the current scoring methodology also implicitly assumes that the levels of cognitive impairment required for answering the ADAS-Cog items incorrectly are uniformly ordered. However, the difficulty levels of the ADAS-Cog items are not uniform [2–4] and most of the total ADAS-Cog scores can actually be achieved by different patterns of scores across the ADAS-Cog items [15]. Moreover, since the ADAS-Cog items vary in their sensitivity to measure the underlying cognitive domains [2–4, 11–13, 15], an item-level analysis is expected to yield better accuracy in measuring cognitive impairment. An item-level analysis is also significant for addressing psychometric problems of the ADAS-cog (such as item bias due to patient factors), which were not investigated at the time of its design [16]. The current scoring methodology does not allow adjustments for such item-level biases, which leads to unaccounted inter-patient variability and further complicates the detection of treatment effects in clinical trials. A related concern pertains to clinical trials that allow inclusion of patients undergoing symptomatic therapy using acetylcholinesterase inhibitor (AChEI) drugs. AChEI drugs provide short term improvements in cognitive performance, specifically in memory-related tasks [17]. If AChEI drugs improve performance on only a subset of the ADAS-Cog items, an item-level analysis may become necessary for isolating the effects of investigative treatments in clinical trials.

Thirdly, the current scoring methodology violates core assumptions of the statistical methods typically employed in clinical trials. The primary efficacy analysis of treatments typically involves linear modeling of serial determinations of the total ADAS-Cog scores of patients using an analysis-of-covariance (ANCOVA) methodology [18–22]. It is reasonable to assume that a patient’s true underlying cognitive impairment progresses linearly over short follow-up durations that are typically considered in clinical trials. However, when cognitive impairment is estimated using the total ADAS-Cog scores, linear modeling using the ANCOVA methodology results in correlated errors due to the categorical nature of the ADAS-Cog items [23, 24]. The ANCOVA methodology assumes errors to be independent and normally distributed, which is violated when the total ADAS-Cog scores are used and results in biased efficacy analysis in trials.

Fourthly, the current scoring methodology lacks a proper definition for the measurement scale of cognitive impairment. This makes comparison and interpretation of cognitive impairment across patients challenging when different variants of the ADAS-Cog are used. In theory, the administration of additional items should only improve measurement precision. However, the current scoring methodology also changes the scale of measurement, with a wider range of scores possible when additional items are administered. The current scoring methodology is also sensitive to missing item responses, scoring errors and variability in the administration of the ADAS-Cog, which are common in clinical trials [25, 26].

In this study, we investigated the hypothesis that addressing these limitations associated with the current scoring methodology would improve the sensitivity of the ADAS-Cog in clinical trials. This resulted in a new scoring methodology for the ADAS-Cog based on a comprehensive psychometric analysis using item response theory (ADAS-CogIRT). Some prior studies have investigated the potential of item response theory for scoring the ADAS-Cog and reported very promising preliminary results [15, 27]. The ADAS-CogIRT methodology is based on extending this prior work, addressing its limitations, and developing a clinically meaningful scale to measure cognitive impairment. We evaluated the sensitivity of the ADAS-Cog using the ADAS-CogIRT methodology and compared it with the current scoring methodology for detecting treatment effects in clinical trials using simulation experiments and data from a real negative clinical trial [18].

## Methods

### Data

The data for this study were assembled from three major public cohorts to ensure that the developed scoring methodology is robust against heterogeneity in patients and study designs. The three cohorts are the Alzheimer’s Disease Neuroimaging Initiative (ADNI), the Coalition against Major Diseases (CAMD), and the Alzheimer’s Disease Cooperative Study (ADCS). These cohorts are briefly described in the Additional file 1: Supplementary Materials. We obtained data from 1,275 participants in ADNI, which included 342 patients clinically diagnosed with probable Alzheimer’s disease at baseline, 866 patients diagnosed with amnestic mild cognitive impairment at baseline, and 67 normal controls who converted to amnestic mild cognitive impairment during follow-up. The clinical dementia rating (CDR) scale was used to select mild cognitively impaired patients in ADNI who are likely to be amnestic type. A global CDR score of 0.5, with at least a 0.5 in the memory domain, was required for inclusion of mild cognitively impaired patients in this study. Out of the 866 mild cognitively impaired patients that satisfied this criterion, 262 converted to a clinical diagnosis of probable Alzheimer’s disease during the course of the study [28]. We additionally collected data from 1,828 Alzheimer’s patients in the placebo arms of six clinical trials in CAMD and 2,496 Alzheimer’s patients in the placebo and treatment arms of six clinical trials in ADCS cohorts. The global CDR scores of Alzheimer’s patients across the three cohorts were approximately uniformly distributed between 0.5 (very mild stage), 1 (mild stage), and 2 (moderate stage) points, resulting in good patient heterogeneity with respect to disease severity.

The data consist of longitudinal ADAS-Cog responses over the duration of trial, basic demographics, apolipoprotein-E (APOE) genotype, and details on concomitant treatments of patients. The most common version of the ADAS-Cog, which contains a ‘Delayed word recall’ item in addition to the original 11 items, was used in this study [1, 29]. Table 1 summarizes the data from the ADNI and the 12 clinical trials of the CAMD and ADCS cohorts. The data were divided into two subsets. The first subset was used for a comprehensive psychometric analysis of the ADAS-Cog and contained data from the ADNI and the placebo arms of all clinical trials except the trial of huperzine A [18]. For psychometric analysis, data from a single visit of every patient was randomly selected to avoid correlated ADAS-Cog responses. The second subset was used to evaluate the scoring methodology we describe in this paper and contained data from the treatment arms of 11 clinical trials. In addition, the clinical trial of huperzine A, which detected a marginally significant treatment effect [18], was used exclusively to evaluate the sensitivity of the new scoring methodology in a real clinical trial scenario.

**Table 1:** Data description: summary of patient characteristics from the ADNI and the clinical trials of the CAMD and ADCS cohorts

Study	Sample size	Gender (% Females)	APOE (% *ε*4 positive)	ADAS-Cog^a^	Study duration
ADNI	1275	41.7	58.7	14.2 ± 8.5	8 years
CAMD-1105	325	51.0	-	25.2 ± 12.2	20 months
CAMD-1131	57	59.6	-	20.5 ± 3.6	24 weeks
CAMD-1132	412	43.4	38.0	19.1 ± 3.1	51 weeks
CAMD-1140	137	42.3	-	19.1 ± 3.4	24 weeks
CAMD-1141	492	55.3	-	9.9 ± 6.0	23 months
CAMD-1142	405	56.0	64.1	25.3 ± 10.4	18 months
ADCS-HU [18]	210	64.4	65.2	27.1 ± 10.8	24 months
ADCS-DHA [19]	402	52.5	57.7	23.9 ± 9.0	18 months
ADCS-VN [20]	300	63.1	71.3	30.1 ± 9.8	24 months
ADCS-HC [21]	409	53.9	70.0	22.6 ± 8.6	18 months
ADCS-LL [22]	406	59.9	55.3	23.9 ± 10.5	18 months
ADCS-MCI [67]	769	47.0	53.0	11.03 ± 4.2	26 months

*ADNI* Alzheimer’s Disease Neuroimaging Initiative, *CAMD* Coalition against Major Diseases, *ADCS* Alzheimer’s Disease Cooperative Study, *APOE* apolipoprotein-E, *HU* Huperzine, *DHA* Docosahexaenoic Acid, *VN* Valproate Neuroprotection, *HC* Homocysteine, *LL* Simvastatin, *MCI* Mild Cognitive Impairment

^a^Summary total ADAS-Cog scores are represented as mean ± standard deviation

The patient data from the ADNI, ADCS, and CAMD cohorts were deidentified before transfer to The University of Texas at Austin. A study specific protocol for the collection and the analysis of deidentified data in this research was approved by the Institutional Review Board of The University of Texas at Austin. Since the data are publicly available and deidentified, ethics approval was not necessary from all the participating institutions in the ADNI, ADCS, and the CAMD cohorts for conducting this research. However, as part of the study protocols for data collection in the three cohorts, all participating institutions obtained ethics approval from their respective institutional review boards in accordance with the Good Clinical Practice guidelines, the Declaration of Helsinki, US 21CFR Part 50-Protection of Human Subjects, and Part 56-Institutional Review Boards, and pursuant to state and federal HIPAA regulations. Written informed consents were obtained from all subjects and authorized study partners in accordance with local institutional review board guidelines before data collection and study-specific procedures were conducted. A list of participating institutions that obtained ethics approval is provided in the Acknowledgements section.

### Psychometric analysis of the ADAS-Cog

We used multidimensional item response theory (IRT) to evaluate the psychometric properties of the ADAS-Cog for measuring cognitive impairment over the course of disease progression. Traditionally, IRT has been employed for investigating psychometric properties of scales in social and educational research that measured a single latent trait in respondents. However, with advances in estimation theory [30–32] and computational abilities, multidimensional IRT models have started to gain popularity since most psychological constructs are unavoidably multidimensional in nature [33]. Using IRT, probabilities of patients’ responses to the ADAS-Cog items were modeled as functions of patients’ underlying cognitive impairment. In psychometric theory, these functions are typically known as the item characteristic functions [33, 34]. Based on the nature of the ADAS-Cog items, patients’ responses are recorded as either dichotomous (response as either correct or incorrect) or ordinal (responses rated on Likert scales). The three-parameter logistic (3PL) model was used to model the probability of an incorrect response to the dichotomous ADAS-Cog items, which included lower asymptotes to account for really difficult items [33, 34]. For instance, if an ADAS-Cog item is difficult and is answered incorrectly by a quarter of cognitively normal individuals, the lower asymptote of that ADAS-Cog item will be estimated as 0.25. On the other hand, the different response categories of the ordinal ADAS-Cog items were modeled using the Samejima’s graded response model [35]. In the Samejima’s graded response models, boundaries between the consecutive response categories are probabilistically modeled using the two-parameter logistic (2PL) models, which can be subtracted to obtain probabilistic models of individual response categories [35]. The key parameters of the ADAS-Cog item characteristic functions are the item slopes and the item intercepts, which represent important characteristics of the ADAS-Cog items. While the slope represents the sensitivity of an item in discriminating between patients with different levels of cognitive impairment, the item intercept represents the difficulty level of an item (or difficulty levels of different response categories of an item) for Alzheimer’s patients. The parameters of the ADAS-Cog item characteristic functions were estimated using the Metropolis-Hastings Robbins-Monro algorithm during both the exploratory [31] and the confirmatory phases of IRT analysis [32].

#### Cognitive domains assessed by the ADAS-Cog

The evaluation of the cognitive domains assessed by the ADAS-Cog in Alzheimer’s patients is important not only for its associated clinical significance but also for ensuring the validity of IRT analysis. IRT makes a strong assumption of local item independence, i.e., patients’ responses to the ADAS-Cog items are determined solely by their underlying extents of cognitive impairment. The use of an inappropriate set of latent traits violates this key assumption, which severely compromises the validity of inferences from IRT analysis [36]. More importantly, local item dependence results in unreliable estimates of latent traits [36], which have been suggested as more accurate measures of cognitive impairment [15, 27]. Therefore, the use of an appropriate number of latent traits is crucial for an accurate IRT-based psychometric analysis of the ADAS-Cog and estimation of latent traits. For this reason, we first performed a parallel analysis on pair-wise polychoric correlations between the ADAS-Cog item responses [37, 38] to determine an upper limit on the number of latent traits to be considered for a more in-depth evaluation [39]. Exploratory IRT models were developed for competing latent trait structures with the number of latent traits ranging from one (unidimensional trait structure) to the upper limit determined by the parallel analysis. No restrictions on item-trait loadings were imposed during the exploratory phase of IRT analysis. For the cases of multidimensional latent trait structures, the item-trait loadings were rotated to oblique solutions (oblimin) with the latent traits allowed to be inter-correlated. The oblique solutions have fewer cross-loadings of items across multiple latent traits, which makes clinical interpretation of latent traits easier. The competing latent trait structures were compared using the following criteria:

1. *Model fit*: The latent trait structure should have good global and item-level fits to the ADAS-Cog responses. Global fit was assessed using the two standard statistics of root mean squared error of approximation (RMSEA) [40] and Tucker Lewis index (TLI) [41]. The criteria of RMSEA ≤ 0.05 and TLI ≥ 0.95 are required for a good global fit [42]. Item-level fit was assessed using the recommended *S-X*^*2*^ statistic, which effectively controls type-I error rates [43, 44].
2. *Local item independence*: The local item independence assumption was tested using the recommended *G*^*2*^ statistic, which has high sensitivity in detecting local item dependence [45].
3. *Clinical relevance*: The individual latent traits should be clinically meaningful constructs that are worth measuring separately. The latent trait structure should be in agreement with the motivation behind the design of the ADAS-Cog items in the original study [1]. Moreover, the latent trait structure should also be supported by the current knowledge of the pathological processes underlying Alzheimer’s disease.

After determining the most appropriate latent trait structure, a confirmatory IRT model was estimated with a restricted item-trait loadings structure. From the exploratory IRT model corresponding to the most appropriate latent trait structure, only the item-trait loadings greater than 0.2 were allowed in the confirmatory IRT model [46]. Furthermore, for the ADAS-Cog items that cross-loaded on multiple latent traits, the weaker item-trait loadings that were less than 0.3 were included only if they significantly improved the model fit of the ADAS-Cog items. The model fit and the validity of the local independence assumption were evaluated for the confirmatory IRT model. The confirmatory IRT model was used for subsequent psychometric analysis of the ADAS-Cog.

#### Measurement invariance of the ADAS-Cog items

The ADAS-Cog items should show measurement invariance across patients, despite their characteristics. We performed differential item functioning (DIF) [47] analyses to investigate measurement bias in the ADAS-Cog items due to the patient-level factors of gender (men/women), education level (less/greater than 13 years), and APOE-*ε*4 genotype (presence/absence of an *ε*4 allele). The ADNI, CAMD, and ADCS cohorts contain predominantly non-Hispanic Caucasian patients, which did not allow DIF analysis due to racial and ethnic factors. In the DIF analyses, the ADAS-Cog item parameters were estimated separately for patient groups and compared using the Lord’s Wald test [34] with the Benjamini and Hochberg false discovery rate correction [48]. The Lord’s Wald test was used instead of the traditional likelihood ratio test [49] due to the large number of hypothesis being tested in this study, which makes the likelihood ratio test very computationally intensive. Using large sample sizes as considered in this study, the Lord’s Wald test has been shown to be sensitive in detecting measurement invariance and asymptotically equivalent to the likelihood ratio test [50].

We additionally investigated the invariance properties of the ADAS-Cog item characteristic functions with respect to the status of concomitant symptomatic therapy using AChEI drugs (presence/absence). In clinical trials involving heterogeneous patient samples with respect to the status of AChEI drugs, the effects of AChEI drugs should be accounted for during statistical analysis to isolate and accurately evaluate the effects of investigative treatments. If AChEI drugs uniformly affect all the ADAS-Cog items assessing a cognitive domain (such as memory), the inclusion of an interaction term with the corresponding progression rate is sufficient to account for the effects of symptomatic therapy. However, an item-level analysis may become necessary if AChEI drugs affect only a subset of the ADAS-Cog items that measure a cognitive domain. In the absence of an item-level analysis, the treatment effects of the AChEI drugs may be inaccurately modeled leading to a biased evaluation of the investigative treatments in clinical trials. While measurement invariance of the ADAS-Cog item characteristic functions is investigated using the same DIF methodology as discussed earlier, violation of measurement invariance should not be interpreted as measurement bias of the ADAS-Cog items but rather as treatment effects of the AChEI drugs on specific subdomains within the cognitive domains.

Longitudinal invariance of the item characteristic functions across different disease stages was also investigated by comparing item parameters estimated using baseline responses of patients versus using their responses at the 24-month visit, when the disease has significantly progressed. We additionally investigated the extent of sample bias and variance in the ADAS-Cog item characteristic functions due to different patient samples considered for estimation. Sample bias was assessed as the goodness-of-fit of the item characteristic functions to the ADAS-Cog response data from the treatment arms of ADCS studies, which were not used for parameter estimation. Sample variance was evaluated by conducting 1,000 bootstrap replications of estimation of the item characteristic functions with sample replacement.

### Measurement of cognitive impairment in patients

#### ADAS-Cog scoring methodology based on IRT modeling (ADAS-CogIRT)

We propose a new ADAS-Cog scoring methodology based on psychometric modeling using IRT (ADAS-CogIRT) for more accurate measurement of cognitive impairment. Given a patient’s responses to the ADAS-Cog items, the ADAS-CogIRT methodology collectively uses the ADAS-Cog item characteristic functions to measure cognitive impairment via maximum-likelihood estimation, i.e., the latent trait values that have the highest likelihood of producing the observed set of item-wise responses. Based on the DIF analysis, appropriate adjustments were included in the item slopes and the intercepts to ensure measurement invariance across patient characteristics. By default, the latent traits in IRT are estimated with means of zero and standard deviations of one. We defined appropriate measurement scales for cognitive impairment by linearly scaling the latent traits obtained from the maximum likelihood estimation, a technique commonly used in educational testing. The values of the scaling parameters were determined based on fulfilling the following two criteria: (1) scores of cognitive impairment in mild-to-moderate Alzheimer’s patients should be non-negative; and (2) scores of cognitive impairment can be rounded off to the nearest integers without loss of precision.

#### Accuracy of the ADAS-CogIRT methodology for measuring cognitive impairment

Since the ground truth cognitive impairment is unknown, the accuracy of the ADAS-CogIRT methodology for measuring cognitive impairment cannot be directly evaluated. Therefore, we indirectly evaluated the ADAS-CogIRT methodology by assessing its accuracy to predict future ADAS-Cog responses of patients based on their responses in a few initial visits. Specifically, we used the ADAS-Cog responses at the baseline, 6-month, and 12-month visits of patients belonging to the treatment arms of the five ADCS studies to obtain longitudinal estimates of their cognitive impairment. These estimates were used to predict cognitive impairment and the corresponding total ADAS-Cog scores at the 24-month visit. The accuracy of the ADAS-CogIRT methodology was calculated using the root mean squared error (RMSE_ADAS_) between the observed and the predicted total ADAS-Cog scores at the 24-month visit. The RMSE_ADAS_ of the ADAS-CogIRT methodology was compared to the RMSE_ADAS_ achieved by using the total ADAS-Cog scores as estimates of cognitive impairment in the initial visits.

#### Precision of the ADAS-CogIRT methodology for measuring cognitive impairment

The precision of the ADAS-CogIRT methodology is dependent on the amount of information contributed by the ADAS-Cog items for measuring different levels of cognitive impairment. We calculated the item information functions of the ADAS-Cog items to estimate the precision of the ADAS-CogIRT scoring methodology [46]. A high value for the item information at a given level of cognitive impairment implies that the item measures that level of cognitive impairment with high precision. Conversely, low item information at a given cognitive impairment level represents that the item measures that level of cognitive impairment with low precision. The composite information across all the ADAS-Cog items was used to estimate the expected standard error of measurement of different levels of cognitive impairment using the ADAS-CogIRT methodology.

### Improving the sensitivity of the ADAS-Cog in clinical trials

#### Application of the ADAS-CogIRT methodology in clinical trials

We propose a generalized mixed-effects approach for using the ADAS-CogIRT methodology in clinical trials. Besides estimating baseline cognitive impairment, this approach estimates the rates of progression in cognitive impairment based on patients’ longitudinal ADAS-Cog responses. We assumed linear progression of cognitive impairment in patients because the durations of clinical trials are typically too short (~2-3 years) to observe any complex patterns of disease progression. Significant inter-patient variability in baseline cognitive impairment and progression rates is typically observed in clinical trials. While some variability is systematic due to patient-level factors (such as APOE-*ε*4 genotype) and treatment effects, random variability across patients is also substantial. Therefore, we modeled baseline cognitive impairment and progression rates as mixed-effects in the model to ensure validity of the key assumptions of efficacy analysis. While the fixed effects modeled systematic variability due to patient factors and treatment effects, the random effects accounted for random variability across patients. We evaluated the sensitivity of the ADAS-CogIRT methodology for detecting treatment effects in clinical trials using simulation experiments and a real clinical trial, which had been reported as negative but which showed some evidence of a treatment effect [18].

#### Sensitivity analysis using clinical trial simulations

Clinical trials were simulated to mimic the complexity of real-world clinical trials by including unbalanced patient samples, systematic and random inter-patient variability in cognitive impairment and progression rates, and dropout of patients from clinical trials. The parameters for simulating these characteristics were obtained by analyzing the longitudinal ADAS-Cog data from the placebo arms of ADCS and CAMD trials using a generalized mixed-effects model approach. A Cox proportional hazards model was used for modeling hazard of patient dropout with baseline cognitive impairment, progression rates, and patient-level factors as covariates.

The statistical power of the newly proposed (ADAS-CogIRT) and the standard ADAS-Cog scoring methodologies for detecting treatment effects was evaluated through two simulation experiments. In the first experiment, their power was evaluated for different sample sizes of 200, 400, 600, 800, and 1,000 patients considered in clinical trials of fixed 24 months duration. For the second experiment, the sample size was fixed as 400 patients and the statistical power was evaluated for different durations of 12, 24, 36, and 48 months. These fixed values were selected based on the average characteristics of past clinical trials. Both experiments were repeated for four hypothetical treatment effects of Cohen’s *d =* 0 (no effect), 0.2 (mild effect), 0.5 (moderate effect), and 0.8 (large effect) simulated in the treatment arms of clinical trials [51]. The case of no treatment effect evaluated the type-I error rates of the proposed scoring methodology. The follow-up visits in both of the experiments were considered to be biannual during the duration of each trial. The ADAS-Cog responses of patients were simulated using the estimated item characteristic functions. The slope and the intercept parameters of the item characteristic functions were randomly perturbed in every trial simulation using the estimated standard errors associated with the parameters. The standard errors represent the expected variability in item parameters if different patient samples were considered for parameter estimation. Therefore, simulating the ADAS-Cog responses using perturbed item parameters resembles real world situations, where patient samples to be analyzed would have different characteristics than the patient sample used for estimating the parameters of the ADAS-CogIRT scoring methodology. The perturbation in the item parameters also reduces the extent of bias involved in our simulation experiments from using the same item characteristic functions for both simulating and analyzing the ADAS-Cog response data.

The simulated ADAS-Cog responses were analyzed using the proposed ADAS-CogIRT and the currently employed analysis-of-covariance (ANCOVA) methodologies. The treatments effects in both of the scoring methodologies were assessed using the *z*-statistic with a Bonferroni correction for multiple comparisons. While a significance level of *α* = 0.05 was considered for the ANCOVA methodology, the significance level for investigating treatments effects using the ADAS-CogIRT scoring methodology was pre-specified as *α* = 0.05/*m*, where *m* denotes the number of cognitive domains assessed by the ADAS-Cog. In both experiments, 500 clinical trials were simulated for every possible combination of treatment effect*,* sample size, and trial duration. The statistical power was evaluated as the proportion of clinical trials in which a statistically significant treatment effect on patients’ progression rates was detected.

#### Sensitivity analysis using a real clinical trial

Besides simulations, we additionally evaluated the sensitivity of the ADAS-CogIRT methodology in a real clinical trial study of huperzine A [18]. In the original negative trial, the higher dose level of 400 *μg* had a marginal effect (p-value = 0.07) on patients’ cognitive functioning after 16 weeks [18]. Given this trend from the original ANCOVA analysis, we were interested in determining whether a more sensitive methodology would change the significance of the treatment effect on progression rates of cognitive impairment. Therefore, we re-analyzed the data from the placebo and the 400 *μg* huperzine A treatment arms using the ADAS-CogIRT methodology. The sample size was 141 patients across the two arms in the 16-week long trial. Besides statistical significance, we also calculated the size of treatment effects estimated by the ANCOVA and the ADAS-CogIRT methodologies for a comparison of sensitivities.

All data analyses in this study were performed using the R 3.2.1 software environment for statistical computing. A more detailed description of our Methods is provided in the Additional file 1: Supplementary Material published online only.

## Results

### Psychometric analysis of the ADAS-Cog

#### Cognitive domains assessed by the ADAS-Cog

From the parallel analysis we estimated that the upper limit on the required number of latent traits is seven. By comparing the latent trait structures for up to seven latent traits using the criteria defined in the 'Cognitive domains assessed by the ADAS-Cog' section, the three-dimensional latent trait structure was found to be the most appropriate one. All models with the number of latent traits greater than or equal to three showed a good global and item-level fit to the ADAS-Cog response data. Local item dependence (LID) between a set of items typically indicates that the item set measures additional latent traits besides the traits already considered in the model. The three-dimensional trait structure had LID only between a few subitems, which belong to the same ADAS-Cog items. Since subitems within items tend to share item-specific contexts, such LID is expected. To eliminate all LID, seven traits were required, where several traits were measured only by single items indicating an overfit to the ADAS-Cog response data. We investigated the effect of the presence of LID on the item parameter estimates of the three-dimensional latent trait structure. The item parameter estimates of IRT models with three and seven latent traits were very similar, which suggests that LID in the three-dimensional model is negligible and does not affect item parameter estimates. While lower asymptotes were considered in the item characteristic functions of all the dichotomous ADAS-Cog items, only the constructional praxis subitem assessing a patient’s ability to draw a cube was found to have a statistically significant lower asymptote of 20.6 %. Drawing a cube is related to the patients’ spatial visualization ability, which is known to deteriorate with aging [52]. Therefore, these results indicate that even 20.6 % of cognitively normal elderly people make mistakes in drawing a cube. In the confirmatory IRT model, only the ‘Draw a cube’ subitem within the ‘Constructional Praxis’ item was allowed to have a non-zero lower asymptote.

**Fig. 1:**
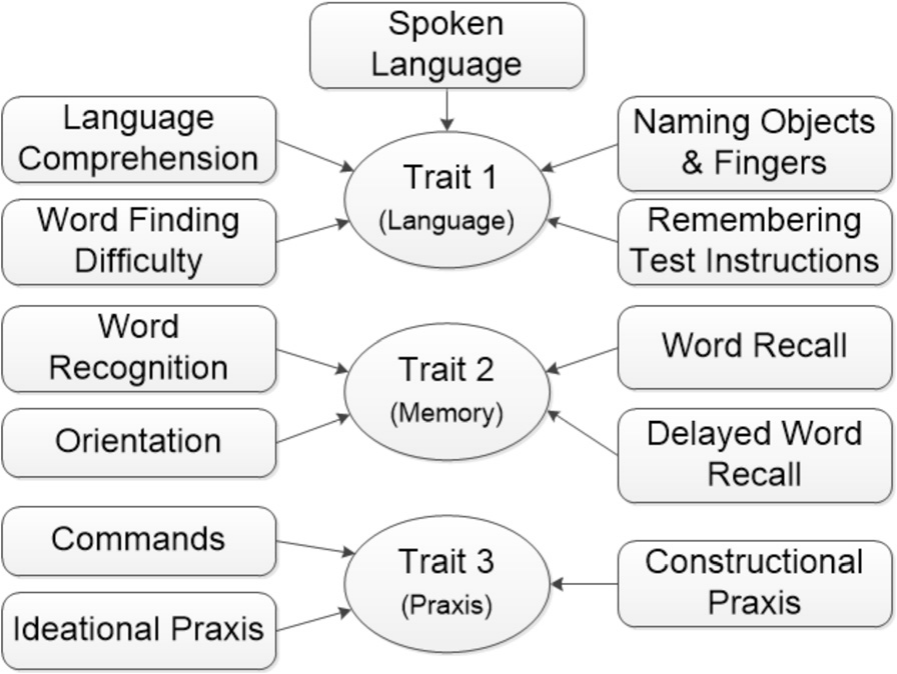
Cognitive domains assessed by the ADAS-Cog: figure showing the item-trait loading structure for the three-dimensional latent trait structure

The three-dimensional trait structure also provides a clinically meaningful interpretation. The pattern of dominant item-trait loadings suggests that the three traits basically represent impairment in the memory, language, and praxis cognitive domains (Fig. 1). In the original study by Rosen et al. [1], the ADAS-Cog items were designed to assess these three cognitive domains with the same associations between the items and the domains as observed in Fig. 1. However, our IRT analysis revealed some additional psychometric properties of the ADAS-Cog items. While the item ‘Remembering test instructions’ was designed to assess the memory domain [1], psychometric analysis suggests that it cross-loads across both the memory and the language domains, with more dominant loading on the language domain. Similarly, the item ‘commands’ was designed to assess the language functions in patients. However, psychometric analysis revealed that it cross-loads between the language and the praxis domains, with more dominant loading on the praxis domain.

The three-dimensional latent trait structure is also supported by the underlying neurodegenerative profile of Alzheimer’s disease. The classic topography of brain tissue loss in Alzheimer’s disease starts early in the medial temporal lobe, which deals with memory functions, followed by involvement of the parietal, frontal, and occipital lobes, which have functions in language processing and praxis [53–56]. A factor analysis of structural brain measurements suggests four distinguishable profiles of neurodegeneration [57], where the brain regions involved in the four profiles are distinctively related to memory, language, and praxis functions. These observations suggest that impairment in the memory, language, and praxis cognitive domains progress differently based on the brain regions involved in different stages of Alzheimer’s disease. Therefore, it would be clinically relevant to separately measure cognitive impairment in the memory, language, and praxis domains. The higher dimensional latent trait structures further divided the memory, language, and praxis domains into several subdomains, which were highly inter-correlated (>0.80). For instance, in the seven dimensional latent trait structure, subdomains of semantic memory and working memory were measured by different latent traits. In view of model conciseness and the high correlations between the memory, language, and praxis subdomains, the three dimensional latent trait structure was found to be the most appropriate for measuring cognitive impairment in Alzheimer’s patients.

The confirmatory IRT model using the item-trait loading structure in Fig. 1 showed good model fit (RMSEA = 0.039, TLI = 0.95, and S-X^2^ insignificant for all the ADAS-Cog items) and low levels of local item dependence as observed in the exploratory IRT model.

#### Measurement invariance of the ADAS-Cog items

Table 2 lists the ADAS-Cog items that violate measurement invariance due to patient-level characteristics. Four ADAS-Cog items have measurement bias due to gender because of different item difficulty for men and women. While naming the object “rattle” is easier for women, they are less likely to correctly name “harmonica” and have more difficulty in drawing a cube. A strong measurement bias due to gender was also observed for the item ‘Remembering test instructions’, where women are more likely to forget test instructions during administration of the ADAS-Cog. No measurement bias was observed due to education level and APOE-*ε*4 genotype. AChEI drugs showed treatment effects only on a subset of the ADAS-Cog items that assess the memory domain. Specifically, the item slopes of the ‘Word recall’, ‘Delayed word recall’, and ‘Word recognition’ items were significantly smaller in patients receiving AChEI drugs. This indicates that patients receiving AChEI drugs have much slower deterioration in their ability to recall and recognize words, which probe short-term working memory. However, other memory-related items (such as ‘Orientation’) assessing other subdomains of the memory domain were not affected by the use of AChEI drugs.

**Table 2:** Differential item functioning: measurement bias of ADAS-Cog items with respect to gender (men/women) and status of concomitant AChEI symptomatic therapy (yes/no)

DIF factor	ADAS-Cog item	Bias type
Gender	Naming objects & fingers: rattle	*d*_*Men*_ < *d*_Women_***
Gender	Naming objects & fingers: harmonica	*d*_*Men*_ < *d*_Women_**
Gender	Constructional Praxis: Cube	*d*_*Men*_ < *d*_Women_**
Gender	Remembering test instructions	*d*_*Men*_ < *d*_Women_***
AChEI	Word recall	*a*_*Yes*_ < *a*_No_***
AChEI	Word recognition	*a*_*Yes*_ < *a*_No_***
AChEI	Delayed word recall	*a*_*Yes*_ < *a*_No_***

*ADAS-Cog* Alzheimer’s disease assessment scale-Cognitive subscale, *AChEI* acetylcholinesterase inhibitors, *DIF* differential item functioning, *d* item intercept/difficulty, *a* item slope

*indicates the level of significance (**for *p*-value <10^-4^ and ***for *p*-value <10^-6^)

The ADAS-Cog item parameters estimated using the baseline and the 24-month visit data did not show any statistically significant differences, which suggests that the ADAS-Cog item characteristic functions are longitudinally invariant. The item characteristic functions also illustrated little sample bias, with good global (RMSEA = 0.039 and TLI = 0.95) and item-level fit (*S-X*^*2*^ was not statistically significant) to response data from the treatment arms of the ADCS clinical trials. The item characteristics functions also showed little variance across different patient samples with a tight agreement observed across 1,000 bootstrap replicates (Additional file 1: Figures S2-S4).

### Measurement of cognitive impairment in Alzheimer’s patients

By default, the parameters of the ADAS-Cog item characteristic functions are estimated such that the scores of memory, language, and praxis impairment have means of 0 and standard deviations of 1 in the patient sample. We found that linear scaling by multiplying with factors of 15 and adding 50 points to the scores of memory, language, and praxis impairment were sufficient for satisfying the two criteria of (1) non-negative cognitive impairment scores for mild-to-moderate Alzheimer’s patients, and (2) standard errors of magnitude ~ 1 point in the mild-to-moderate Alzheimer’s stage. Similar approaches have been previously utilized with different scaling factors in the educational and social domains. Instead of scaling the cognitive impairment scores post-estimation, we performed an equivalent linearly scaling of the ADAS-Cog item parameters to enforce these measurement scales for estimation of memory, language, and praxis impairment. An additional advantage of the defined measurement scales is the fractional interpretation of the cognitive impairment scores. Severe Alzheimer’s patients have cognitive impairment scores close to 100 points and, therefore, a patient’s extent of cognitive impairment can be interpreted fractionally relative to severe Alzheimer’s disease patients, who have lost the ability to independently function in daily life activities.

#### Accuracy of the ADAS-CogIRT methodology for measuring cognitive impairment

**Fig. 2:**
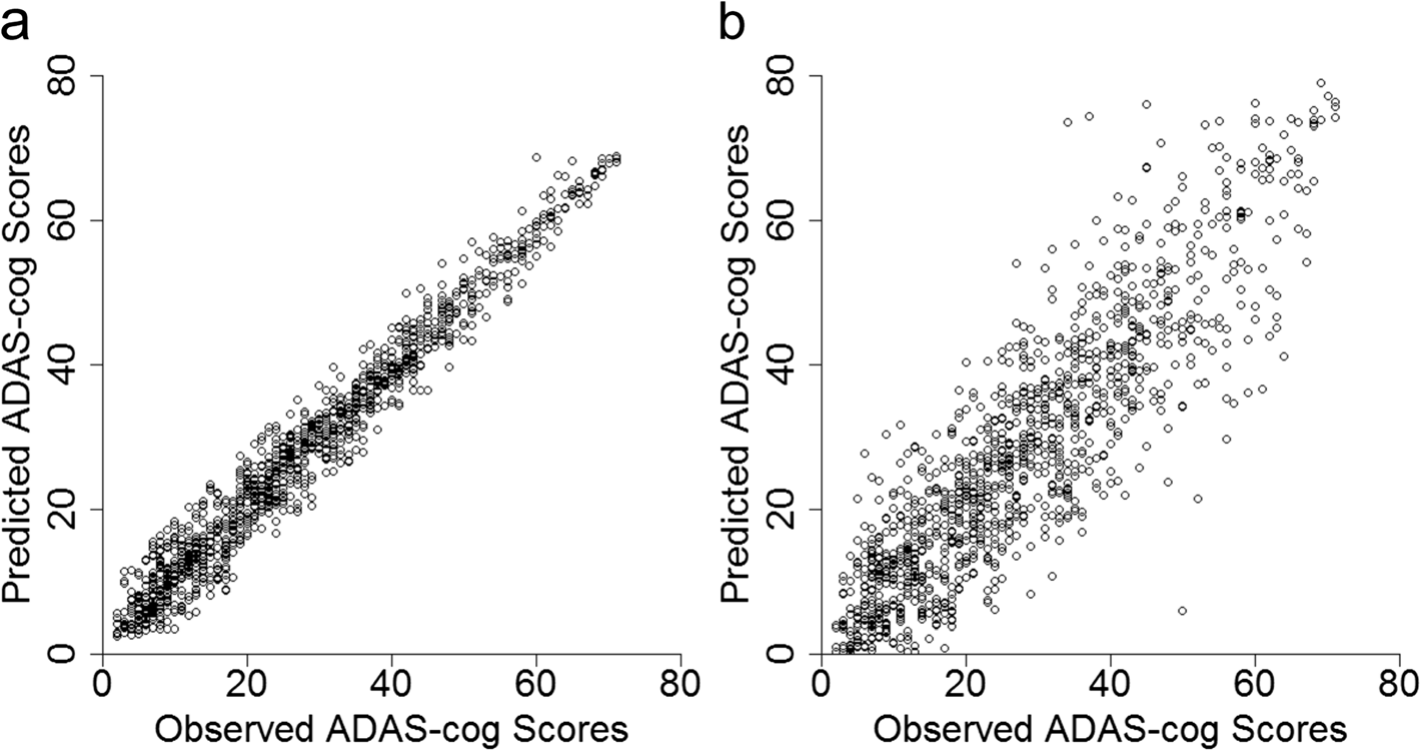
Accuracy of the ADAS-CogIRT methodology: scatterplots showing agreement between the observed total ADAS-Cog scores and the predicted total ADAS-Cog scores at the 24-month visit using **a** the proposed ADAS-CogIRT methodology and **b** the standard scoring methodology. *ADAS-CogIRT* Alzheimer’s disease assessment scale-Cognitive subscale scoring methodology based on item response theory

The ADAS-CogIRT methodology illustrated good accuracy in predicting total ADAS-Cog scores at the 24-month visit with RMSE_ADAS_ = 1.82 points. In comparison, the current scoring methodology resulted in an error of RMSE_ADAS_ = 6.05 points, which is similar in magnitude to the annual change of 5-10 points in the total ADAS-Cog scores of mild-to-moderate Alzheimer’s patients [58, 59]. Figure 2 qualitatively compares the predictive accuracies of the ADAS-CogIRT and the current scoring methodologies.

#### Precision of the ADAS-CogIRT methodology for measuring cognitive impairment

**Fig. 3:**
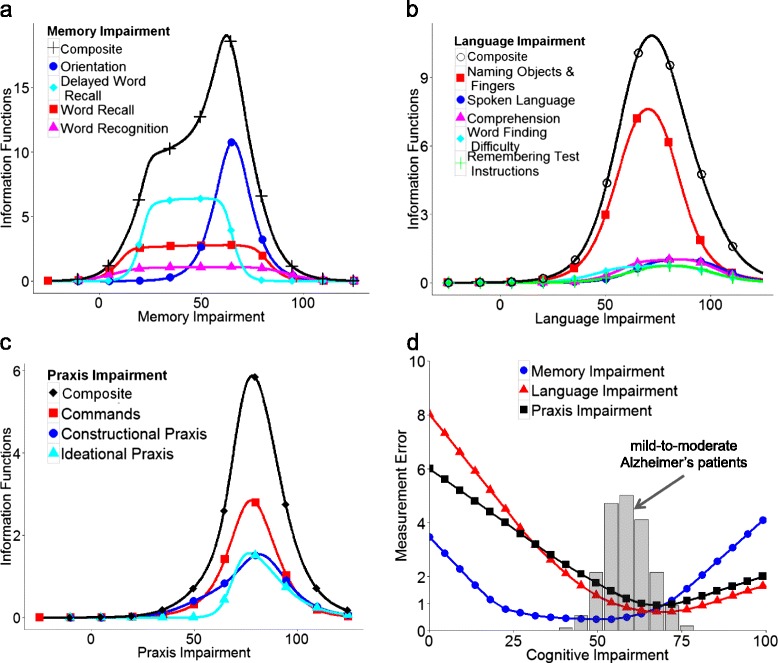
Precision of the ADAS-CogIRT methodology: figure showing item-wise and cumulative Fisher information associated with the estimation of (**a**) memory, (**b**) language, and (**c**) praxis impairment. The plot in (**d**) shows the expected magnitudes of estimation errors associated with different levels of memory, language, and praxis impairment. The superimposed histogram in plot (d) shows the distribution of baseline cognitive impairment in mild-to-moderate Alzheimer’s patients, which have been appropriately scaled for better interpretation. *ADAS-CogIRT* Alzheimer’s disease assessment scale-Cognitive subscale scoring methodology based on item response theory

While the memory items of the ADAS-Cog are informative over the whole range of memory impairment, language and praxis items hold information only for pronounced levels of language and praxis impairment (Fig. 3a-c). The ADAS-CogIRT methodology shows good precision for almost the whole range of memory impairment. However, due to the inherent limitation of the ADAS-Cog items, the precision of the ADAS-CogIRT methodology in measuring language and praxis impairment is good only when a patient’s performance is quite poor (Fig. 3d).

### Improving the sensitivity of the ADAS-Cog in clinical trials

The treatment effects in the simulated trials and the huperzine A trial were investigated using the ADAS-CogIRT scoring methodology at a significance level of *α* = 0.05/3 since treatments are evaluated in the three domains of memory, language, and praxis simultaneously.

#### Sensitivity analysis using clinical trial simulations

**Fig. 4:**
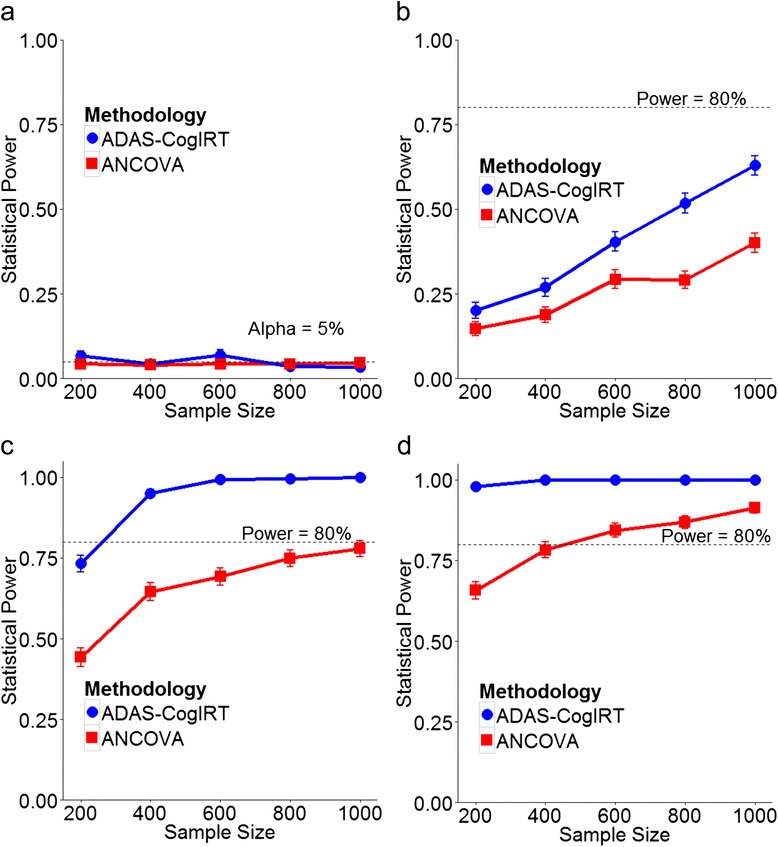
Statistical power against sample size: plots showing the relationship between the statistical power of the ADAS-CogIRT and the ANCOVA methodologies and sample size for hypothetical treatment levels of (**a**) *d = 0*, (**b**) *d = 0.2*, (**c**) *d = 0.5*, and (**d**) *d = 0.8*. The trial duration was fixed at 24 months. *ADAS-CogIRT* Alzheimer’s disease assessment scale-Cognitive subscale scoring methodology based on item response theory, *ANCOVA*, analysis of covariance

**Fig. 5:**
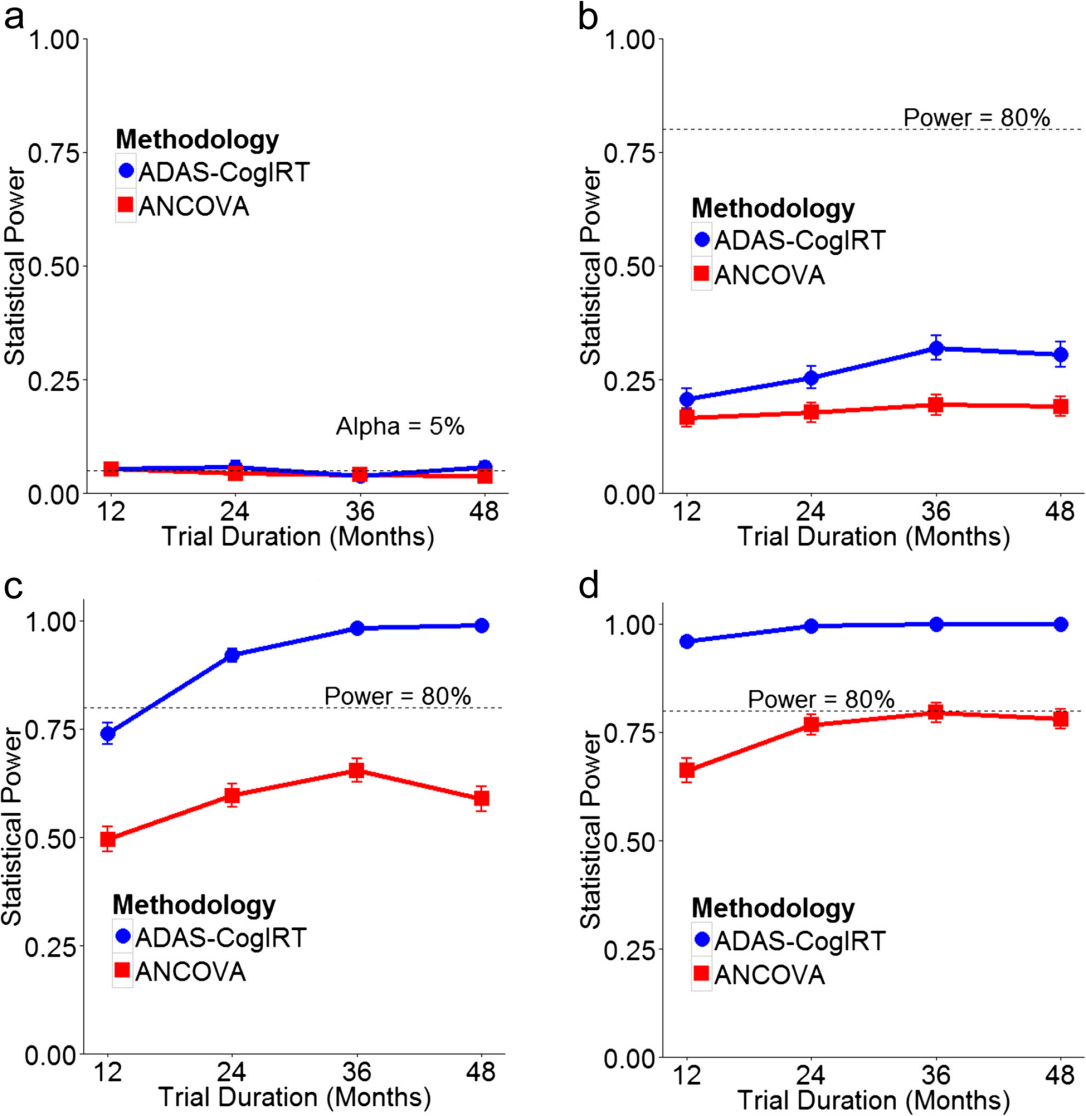
Statistical power against trial duration: plots showing the relationship between the statistical power of the ADAS-CogIRT and the ANCOVA methodologies and duration of clinical trials for hypothetical treatment levels of (**a**) *d = 0*, (**b**) *d = 0.2*, (**c**) *d = 0.5*, and (**d**) *d = 0.8*. The sample size was fixed at 400 patients. *ADAS-CogIRT* Alzheimer’s disease assessment scale-Cognitive subscale scoring methodology based on item response theory, *ANCOVA*, analysis of covariance

In detecting simulated treatment effects, the ADAS-CogIRT methodology provides significant improvements in statistical power over the currently used ANCOVA methodology (Figs. 4b-d and 5b-d). For a mild treatment effect (Figs. 4b and 5b), both methodologies have low power and are unable to attain the 80 % power cut-off even with large sample sizes and long trial durations. This is due to large inter-patient variability in progression rates within each trial arm, which obscures the presence of a mild treatment effect. However, in comparison to the ANCOVA methodology, the ADAS-CogIRT methodology shows better improvements in statistical power as sample size and trial duration are increased (Figs. 4b and 5b). In the case of a moderate treatment effect, the ADAS-CogIRT methodology shows significantly better statistical power than the ANCOVA methodology. The ADAS-CogIRT methodology attains the 80 % power threshold in trials with much smaller sample size (*~*300 patients) and shorter trial duration (*~*18 months) than the ANCOVA methodology, which requires *~* 1,000 patients in a 24-month trial to achieve 80 % power (Fig. 4c). With a sample size of 400 patients, the ANCOVA methodology never achieves 80 % power even if the trial duration is increased to over four years (Fig. 5c). However, the performance of the ANCOVA methodology improves for a large treatment effect (Figs. 4d and 5d). While the ADAS-CogIRT methodology achieves ~100 % power for all sample sizes and trial durations, the ANCOVA methodology also shows good sensitivity reaching 80 % power with *~* 450 patients in a 24-month trial. The improvement in statistical power of both methodologies with an increase in trial duration was less than that observed with an increase in sample size. Both methodologies have acceptable type-1 error rates of ~5 % for different sample sizes and trial durations (Figs. 4a and 5a).

#### Sensitivity analysis using a real clinical trial

The analysis of the huperzine A trial data using the ADAS-CogIRT methodology revealed that 400 *μg* huperzine A reduces the annual progression rate of praxis impairment by 14.75 points/year (*z* = -2.71, *p*-value = 0.0066). The effects of huperzine A on progression rates of memory (*z* = -1.04, *p*-value = 0.30) and language impairment (*z* = -1.63, *p*-value = 0.10) were not statistically significant. The size of the treatment effect detected by the ADAS-CogIRT methodology (*d =* 1.97) was significantly higher than that detected by the ANCOVA methodology (*d =* 0.35). Since praxis items contribute the least to the total ADAS-Cog scores (15/70 points), the ANCOVA methodology detects a much smaller treatment effect in comparison to the ADAS-CogIRT methodology. When only the praxis ADAS-Cog items are used in the current scoring methodology, the ANCOVA analysis detects a significantly larger treatment effect size (*d* = 0.68), which is statistically significant (*z* = -2.49, *p*-value = 0.012).

## Discussion

The proposed ADAS-CogIRT scoring methodology addresses several limitations associated with the current scoring methodology. An in-depth psychometric analysis showed that the ADAS-Cog measures impairment in the three distinct cognitive domains of memory, language, and praxis in patients. This is in agreement with the design of items in the original ADAS-Cog study [1] and findings of several other factor analysis studies [11–13, 60]. While memory loss has been long considered characteristic of Alzheimer’s disease, its classic neuropathology can also be associated with important language and praxis impairment in patients with predominant posterior perisylvian damage [61]. Similar to AChEI drugs, which specifically target memory mechanisms, and to the effect we detected in the huperzine A trial, investigative treatments in the future may also have non-uniform effects across the three cognitive domains. The current scoring methodology cannot detect non-uniform effects across the cognitive domains. In contrast, the ADAS-CogIRT methodology allows for separate evaluation of treatment effects on the memory, language, and praxis domains.

The ADAS-CogIRT methodology estimates cognitive impairment based on patients’ response patterns across the ADAS-Cog items. Such an item-level analysis also allows adjustment for measurement bias of the ADAS-Cog items due to gender. Gender differences in item difficulty are likely due to socio-cultural factors that expose one gender to certain objects and tasks more often than the other gender experiences them. AChEI drugs have a treatment effect on patients’ performance on word recall and recognition items. Since these items contribute heavily to the total ADAS-Cog scores (32/80 points), this may be the reason behind the slower cognitive deterioration observed in patients undergoing AChEI drugs, as assessed by the current methodology [62, 63]. Since only a subset of the ADAS-Cog items assessing the memory domain are affected by the AChEI drugs, adjustments in the item characteristic functions of the three affected ADAS-Cog items are important before application in clinical trials. If such adjustments are not included, the invariance properties of the ADAS-Cog item characteristic functions are violated resulting in an underestimation of cognitive impairment in patients who are taking AChEI drugs. Moreover, the effects of the investigative treatments in clinical trials may be estimated with a positive bias since the treatment effects of the AChEI drugs are underestimated on a domain-level analysis. While including the treatment effects of the AChEI drugs within the ADAS-Cog item characteristic functions may be controversial, we recommend these item-level adjustments in clinical trials since the primary goal is to accurately evaluate the investigative treatments. A domain-level modeling of the AChEI treatment effects is expected to produce biased estimates of the effects of the investigative treatments on the memory domain. Therefore, it makes sense to utilize the established treatment effects of the AChEI drugs within the statistical modeling framework to more accurately investigate the effects of investigative treatments. It should be noted that the experiments presented in this study did not require such item-level adjustments because we did not consider the use of AChEI drugs in simulated clinical trials and the huperzine A trial is homogeneous with respect to AChEI therapy status.

Inspired by the application of IRT in educational testing, we defined a clinically meaningful scale to measure cognitive impairment. In mild-to-moderate Alzheimer’s patients, the scale allows estimates to be rounded off to the nearest integers without loss of precision. The scale also facilitates a fractional interpretation of cognitive impairment in study patients, relative to severely impaired patients, who have a cognitive impairment score of 100 points. The parameters of the ADAS-CogIRT methodology are scale independent. Therefore, items can be easily added or removed from the ADAS-CogIRT methodology without having to re-estimate parameters or redefine properties (such as range) of the measurement scale. This is relevant because active research towards improving the ADAS-Cog items is already underway [9]. Since the ADAS-CogIRT methodology pools information across items for estimating cognitive impairment, it is less sensitive to scoring errors in individual items as compared to the current scoring methodology, which is linearly affected. For patients with missing responses to certain items, the ADAS-CogIRT methodology does not require data imputation and estimates cognitive impairment using the set of items answered by the patients. However, measurement precision is lower for patients with missing responses, as would be expected from psychometric theory.

By addressing limitations of the current scoring methodology, the ADAS-CogIRT methodology measures cognitive impairment more accurately (Fig. 2) and makes clinical trials more efficient by reducing the sample size and the follow-up duration required to investigate treatments (Figs. 4 and 5). More importantly, it allows for the detection of treatment effects that may be missed by using the current scoring methodology. This was validated using data from the huperzine A clinical trial, where the ADAS-CogIRT methodology detected a significant improvement in the praxis domain that had been overlooked using the current scoring methodology. The current scoring methodology obscures detection of effects of treatments that only improve a subset of cognitive domains. This is evident from the observation that when the ANCOVA analysis is repeated using only the praxis ADAS-Cog items in the current scoring methodology, a statistically significant treatment effect is detected. In agreement with these findings, a positive effect of huperzine A on praxis abilities of patients has been found using the activities of daily living scale [64, 65]. It is noteworthy that while we assumed linear progression of cognitive impairment in clinical trials, future studies involving longer durations may require models of nonlinear profiles of progression of cognitive impairment. The presented generalized mixed-effects approach for utilizing the ADAS-CogIRT scoring methodology in clinical trials is flexible and can be extended to include such nonlinear profiles of progression.

Prior work on the application of IRT to the ADAS-Cog mostly focused on evaluating its measurement properties [4, 60, 66]. A few studies additionally investigated IRT for measuring cognitive impairment [15, 27]; however, they assumed that the ADAS-Cog measures a single trait in patients. While a single trait is easy to interpret and model using IRT, it does not adequately fit patient response data (Additional file 1: Figures S1a-b) and severely violates the core IRT assumption of local item independence, which has severe effects on trait estimates [36]. Similar to the total ADAS-Cog scores, the single trait also measures a weighted average of impairment across multiple cognitive domains. Memory items, which have the highest weights, show the poorest fit to the ADAS-Cog response data (Additional file 1: Figure S1b). As a result, measurement of cognitive impairment from a single latent trait IRT model suffers from low precision and reliability. Despite these shortcomings, a single trait IRT model has been demonstrated to significantly improve the sensitivity of the ADAS-Cog in clinical trial simulations [27]. However, those reported results may be overly optimistic because several of the trial characteristics simulated in the analysis [27] are atypical for real clinical trials, such as frequent follow-ups, no patient dropouts, and no heterogeneity due to patient-level factors. Therefore, for a proper comparison, we additionally evaluated the single trait version of the ADAS-CogIRT methodology in more realistic clinical trial simulations and found it to illustrate significantly lower power than the proposed ADAS-CogIRT methodology (Additional file 1: Figures S5 and S6). Since prior studies were primarily focused on evaluating the potential of IRT in this application domain, they did not define a measurement scale [15, 27], resulting in counterintuitive negative scores of cognitive impairment in study patients. As also noted by the authors [15, 27], they were additionally limited by ignoring measurement bias and heterogeneity in disease severity of patients.

While our study addressed several limitations of the current scoring methodology, some limitations persist. Firstly, we could not investigate measurement invariance of the proposed scoring methodology across all patient-level factors (such as race and ethnicity) due to a lack of heterogeneity in the data. This limitation should be noted in future work in order to avoid biased estimates of cognitive impairment using the ADAS-CogIRT methodology with patient groups not considered in this study. Secondly, when compared to the current scoring methodology, the ADAS-CogIRT methodology requires the use of a computer or a handheld device for measuring cognitive impairment in patients. However, this limitation is less relevant for clinical trials than for routine practice because computing is already required for efficacy analysis of investigative treatments. For routine practice, a specialized application (e.g., for a tablet or phone) could be developed to help providers use the ADAS-CogIRT methodology. Thirdly, the precision of the ADAS-CogIRT methodology for measuring language and praxis impairment is affected by the inherent limitations of the ADAS-Cog items (Fig. 3). As a result, the improvement in sensitivity afforded by the ADAS-CogIRT methodology will decrease for clinical trials focusing on milder stages of Alzheimer’s disease. In those disease stages, it may be better to use this tool only for investigating treatment effects on memory impairment. However, this approach would not be applicable to mild Alzheimer’s disease patients who have predominant involvement of the parietal lobe [61]. The inclusion of more difficult items probing subtle levels of language and praxis impairment would improve its measurement precision in milder stages of Alzheimer’s disease. Fourthly, as is the case for most simulation studies, the evaluation results using simulated clinical trials suffer from some bias. The bias is primarily because the estimated item parameters are used for both simulating patients’ response data and within the ADAS-CogIRT scoring methodology for detecting treatment effects. While we reduced the bias by perturbing the ADAS-Cog item parameters (using their estimated standard errors) before simulating patients’ ADAS-Cog responses, some bias is still expected.

Despite these limitations, the ADAS-CogIRT methodology holds great significance for clinical trials of Alzheimer’s treatments. A significant proportion of clinical trials still focus on the mild-to-moderate disease stages due to the inability to detect Alzheimer’s disease early with high specificity. The proposed scoring methodology significantly improves the efficiency of clinical trials focused on the mild-to-moderate stages of Alzheimer’s disease. Such an improvement in efficiency of clinical trials is highly desirable for rapid testing of future treatments in the critical quest for a disease-modifying treatment. The ADAS-CogIRT methodology also allows separate evaluation of treatment effects in the memory, language, and praxis domains, which can potentially provide additional information on the pharmacological properties of treatments and facilitate development of improved therapies. Future clinical trials of Alzheimer’s treatments should consider the proposed ADAS-CogIRT scoring methodology as part of their secondary efficacy analysis to further evaluate and establish the significance of the proposed methodology in comparison to the current scoring methodology.

## Conclusions

The sensitivity of the Alzheimer’s disease assessment scale-cognitive subscale (ADAS-Cog) in its current form can be significantly improved by addressing limitations associated with its scoring methodology. In this study, we described a new scoring methodology for the ADAS-Cog called the ADAS-CogIRT, which addresses several major limitations of the current scoring methodology and significantly improves the sensitivity of the ADAS-Cog in measuring progression in cognitive impairment in clinical trials. Future clinical trials of Alzheimer’s disease-modifying treatments should consider the application of the described scoring methodology as part of their secondary efficacy analysis to further validate its significance in comparison to the currently employed scoring methodology.

## Additional file

Additional file 1: **Supplementary materials: A supplementary document provides details on the methods required for reproducing the results reported in this paper.** The supplementary material also contains some additional statistical results, which have not been included in the paper. (PDF 2056 kb)

## Abbreviations

2PL: 2 parameter logistic
3PL: 3 parameter logistic
AChEI: Acetylcholinesterase inhibitors
ADAS-Cog: Alzheimer’s disease assessment scale-Cognitive subscale
ADAS-CogIRT: ADAS-Cog scoring methodology based on item response theory
ADCS: Alzheimer’s Disease Cooperative Study
ADNI: Alzheimer’s Disease Neuroimaging Initiative
ANCOVA: Analysis of covariance
APOE: Apolipoprotein-E
CAMD: Coalition Against Major Diseases
CDR: Clinical dementia rating
DIF: Differential item functioning
IRT: Item response theory
LID: Local item dependence
RMSEA: Root mean squared error of approximation
TLI: Tucker Lewis index
