# Supplementary material for: New scoring methodology improves the sensitivity of the Alzheimer’s Disease Assessment Scale-Cognitive subscale (ADAS-Cog) in clinical trials
Source: Alzheimers Res Ther. 2015 Nov 12;7:64. doi: 10.1186/s13195-015-0151-0 (PMC4642693; doi:10.1186/s13195-015-0151-0)
Supplement: Supplementary file 1 — Supplementary materials: A supplementary document provides details on the methods required for reproducing the results reported in this paper. The supplementary material also contains some additional statistical results, which have not been included in the paper. (PDF 2056 kb) [file 13195_2015_151_MOESM1_ESM.pdf]

Supplementary material

New scoring methodology improves the sensitivity of the Alzheimer's Disease Assessment Scale-Cognitive subscale (ADAS-Cog) in clinical trials

Nishant Verma, S. Natasha Beretvas, Belen Pascual, Joseph C. Masdeu, Mia K. Markey

Abstract

This supplementary document provides a more detailed description of the methods and some additional results, which were not presented in the paper.

1. Data Description

A brief description of the Alzheimer's Disease Neuroimaging Initiative (ADNI), the Coalition Against Major Diseases (CAMD), and the Alzheimer's Disease Cooperative Study (ADCS) datasets is as follows:

1. **ADNI**: The ADNI was launched in 2003 as a collaboration between several private and public institutions including the National Institute on Aging (NIA), the National Institute of Biomedical Imaging and Bioengineering (NIBIB), and the Food and Drug Administration (FDA). The primary goal of ADNI has been to test whether medical imaging, biological markers, clinical and neuropsychological assessments can be combined to measure the progression of mild cognitive impairment (MCI) and early Alzheimer's disease (AD). The subjects in ADNI have been recruited from over 50 sites across the U.S. and Canada.

2. **CAMD**: The Critical Path Institute, in collaboration with the Engelberg Center for Health Care Reform at the Brookings Institution, formed the Coalition Against Major Diseases (CAMD) in 2008. The Coalition brings together patient groups, biopharmaceutical companies, and scientists from academia, FDA, the European Medicines Agency (EMA), the National Institute of Neurological Disorders and Stroke (NINDS), and NIA. The data available in the CAMD database were volunteered by CAMD member companies and non-member organizations. CAMD database contains de-identified control arm data on AD patients from 24 clinical trials of disease-modifying treatments.

3. **ADCS**: The ADCS is a major initiative for Alzheimer's disease clinical studies, developed as a cooperative agreement between the NIA and the University of California, San Diego in 1991. The goal of ADCS is to facilitate discovery, development and testing of new treatments for Alzheimer's disease. Since 1991, ADCS has initiated 30 research studies (23 drug studies and 7 instrumental development protocols) over 20 Alzheimer's disease research centers.

2. ADAS-Cog Summary & Preprocessing

We used the most common version of the ADAS-Cog, which contains a 'Delayed word recall' item in addition to the original eleven items. Out of the twelve items, five items ('Naming objects and fingers', 'Commands', 'Constructional praxis', 'Ideational praxis', and 'Orientation') contain several subitems such as 'Draw a cube'. Instead of combining the subitem scores, we analyzed these five items at their subitem-level as dichotomous items. The remaining ADAS-Cog items have ordinal responses and were considered as polytomous items for item response theory modeling.

Several items in the ADAS-Cog suffer from severe floor and ceiling effects, which are difficult to model using item response theory. Therefore, as part of the preprocessing step, items with $<5\%$ incorrect response rate from mild-to-moderate Alzheimer's patients were either combined with other similar items or removed from the analysis. In the 'Naming objects and fingers' item, all the high frequency objects ('Flower', 'Bed', 'Whistle', and 'Pencil') were combined into a single subitem called the 'High frequency objects'. While the 'Wallet' object is listed as a low frequency object, its incorrect response rate matched with the rates of the medium frequency objects. Therefore, the objects 'Scissors', 'Comb', and 'Wallet' were combined into a single subitem called the 'Medium frequency objects'. The subitem requiring patients to name the finger 'Thumb' was removed from the analysis due to very low incorrect response rate.

In the 'Commands' item, the subitems 'Point to the ceiling, then to the floor' and 'Put the pencil on top of the card, then put it back' were combined into a 'Easy commands' subitem. Similarly, in the 'Constructional praxis' item, the subitems requiring patients to draw a 'Circle' and 'Two overlapping rectangles' were combined into a 'Easy constructional praxis' subitem. The subitems 'Fold a letter', 'Put letter in envelope', and 'Seal envelope' have low incorrect response rates and, therefore, were combined into a single 'Easy ideational praxis' subitem. The subitem asking patients to recall their 'Full name' has very low incorrect response rate and, therefore, was removed from the analysis. For the ordinal items 'Language', 'Comprehension of spoken language', 'Word finding difficulty', and 'Remembering test instructions', the 'Severe' response category was merged with the 'Moderately severe' response category.

3. Psychometric Analysis of the ADAS-Cog

3.1. Item Response Theory (IRT)

Patients' responses to the ADAS-Cog items were probabilistically modeled by defining ADAS-Cog item characteristic functions, which specify relationships between the characteristics of the ADAS-Cog items (item slope and item intercept) and characteristics of the patients (extent of cognitive impairment). For the sake of understanding the underlying motivation behind defining item characteristic functions, lets consider the case of a dichotomous item with possible responses as either a correct response or an incorrect response. The extent of cognitive impairment can be considered as a continuous measure such that as cognitive impairment increases, the ability to answer an item correctly decreases. The ability of an item to discriminate between two different levels of cognitive impairment can be considered as one of the characteristics (slope) of the items such that an item with higher slope will have better sensitivity in detecting fine differences in cognitive impairment. Another characteristic of the items can be a threshold value of cognitive impairment (or difficulty/location as frequently used in IRT) such that patients with more pronounced levels of cognitive impairment than the threshold would have higher chances of answering the item incorrectly than answering it correctly. By using these two characteristics of the item, a relationship (item characteristic function) can be defined between a patient's cognitive impairment and probability of an incorrect response to the item.

Mathematically, for a dichotomous ADAS-Cog item j with response categories as $x_{\cdot j} \in \{0, 1\}$, the item characteristic function relating the probability of an incorrect response $x_{ij} = 1$ by patient i with cognitive impairment $\boldsymbol{\theta_i}$ was defined as:

$$P(x_{ij} = 1 | \boldsymbol{\theta_i}, \boldsymbol{\alpha_j}, d_j, g_j) = g_j + \frac{(1 - g_j)}{1 + \exp[-(\boldsymbol{\alpha_j^T} \boldsymbol{\theta_i} + d_j)]} \tag{1}$$

where, $\boldsymbol{\theta_i} = (\theta_{i1}, \ldots, \theta_{im})$ denotes a vector of impairment in the m cognitive domains that are assessed by the ADAS-Cog, $\boldsymbol{\alpha_j} = (\alpha_{j1}, \ldots, \alpha_{jm})$ are the item slope components associated with impairment in the m cognitive domains, and d_j is the item intercept. The item intercept d_j represents the relative difficulty level of the item j in comparison to rest of the ADAS-Cog items. The lower asymptotes g_j were included to account for really difficult items, which are answered incorrectly even by cognitively normal individuals.

The definition of item characteristic function for the dichotomous ADAS-Cog items in equation (1) was extended to the polytomous ADAS-Cog items with $C_j \geq 2$ response categories $x_{\cdot j} \in \{0, \ldots, C_j - 1\}$ by modeling the boundaries

between the response categories as

$$
\begin{aligned}
P(x_{ij} \geq 0|\boldsymbol{\theta_i}, \boldsymbol{\alpha_j}, \boldsymbol{d_j}) &= 1, \\
P(x_{ij} \geq 1|\boldsymbol{\theta_i}, \boldsymbol{\alpha_j}, \boldsymbol{d_j}) &= \frac{1}{1+\exp[-(\boldsymbol{\alpha_j^T}\boldsymbol{\theta_i}+d_{j1})]}, \\
P(x_{ij} \geq 2|\boldsymbol{\theta_i}, \boldsymbol{\alpha_j}, \boldsymbol{d_j}) &= \frac{1}{1+\exp[-(\boldsymbol{\alpha_j^T}\boldsymbol{\theta_i}+d_{j2})]}, \\
&\vdots \\
P(x_{ij} \geq C_j|\boldsymbol{\theta_i}, \boldsymbol{\alpha_j}, \boldsymbol{d_j}) &= 0
\end{aligned}
$$

where, $\boldsymbol{d_j} = (d_{j1}, \ldots, d_{j(C_j-1)})$ are the intercepts corresponding to the boundaries between the response categories of item j. The item characteristic functions for individual response categories $x_{ij} = k$ of the ADAS-Cog polytomous items were obtained as

$$
\begin{aligned}
P(x_{ij} = k|\boldsymbol{\theta_i}, \boldsymbol{\alpha_j}, \boldsymbol{d_j}) = P(x_{ij} \geq k|\boldsymbol{\theta_i}, \boldsymbol{\alpha_j}, \boldsymbol{d_j}) - \\
P(x_{ij} \geq k+1|\boldsymbol{\theta_i}, \boldsymbol{\alpha_j}, \boldsymbol{d_j})
\end{aligned} \tag{2}
$$

Metropolis-Hastings Robbins-Monro (MHRM) algorithm [1] was used for estimating the ADAS-Cog item parameters $\boldsymbol{\Psi} = \{\boldsymbol{\alpha_j}, \boldsymbol{d_j}; j = 1, \ldots, n\}$ as it is more computationally efficient than the traditional expectation maximization algorithm [2] for estimating multidimensional item response theory models.

3.2. Cognitive domains assessed by the ADAS-Cog

Since all of the ADAS-Cog items have categorical responses, pairwise polychoric correlations were calculated between the ADAS-Cog items and used in parallel analysis for estimating the dimensionality of latent traits assessed by the ADAS-Cog. Parallel analysis estimated the number of latent traits as $m = 7$, where only 5 traits were associated with eigenvalues ≥ 1. This suggested that the parallel analysis overestimated the number of latent traits by even accounting for weak traits measured by small subsets of the ADAS-Cog items. Therefore, the $m = 7$ estimate from the parallel analysis was used only as an upper limit on the number of latent traits to be considered for a more comprehensive psychometric evaluation. Exploratory IRT models were developed with the number of latent traits ranging from $m = 1$ to 7. No restrictions were imposed on the item-trait loadings and the latent traits were allowed to be inter-correlated. The seven latent trait structures were compared based on (i) the goodness-of-model fit, (ii) adherence to the local item independence assumption, and (iii) clinical relevance of the latent traits, as described in the paper.

All latent trait structures with the number of latent traits $m \geq 3$ showed good global fit to the ADAS-Cog response data with RMSEA$\leq$0.05 and TLI$\geq$0.95 (figure S1a). While the unidimensional IRT model showed an acceptable value for RMSEA$\sim$0.05, it failed to illustrate an acceptable global fit with TLI$\sim$0.89.

This misfit is evident from an item-level assessment of model fit, where the unidimensional structure illustrates poor fit to the response data of all the memory-related ADAS-Cog items (figure S1b). While the inclusion of additional latent traits improved the model fit of memory items, the item-level fit did not show any significant improvements after the inclusion of 3 latent traits (figure S1b).

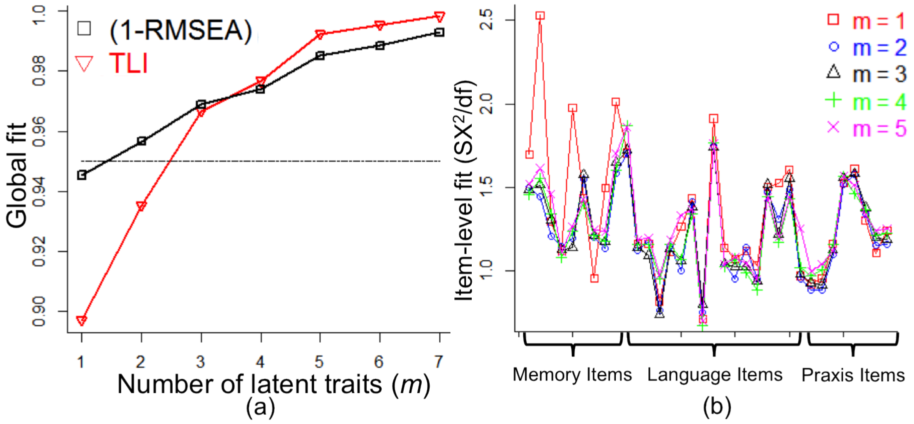

Figure S1: Goodness-of-model fit to the ADAS-Cog response data: Figure comparing (a) global fit and (b) item-level fit of the seven latent trait structures to the ADAS-Cog response data. The black dashed line in subfigure (a) represents the typical cut-off of $RMSEA = 0.05$ and $TLI = 0.95$ for a good model fit. The item-level fit in subfigure (b) did not improve after $m \geq 3$ latent traits and, therefore, the cases of $m \geq 6$ have not been included for clarity of presentation.

We verified the findings of the exploratory IRT analysis by performing a confirmatory IRT analysis on an independent sample of the ADAS-Cog response data. A three-dimensional confirmatory IRT model was developed using the ADAS-Cog response data from the treatment arms of ADCS clinical trials. The confirmatory IRT model showed good global fit (RMSEA = 0.039 and TLI = 0.95), good item-level fit ($S - X^2$ insignificant), and low levels of local item dependence between subitems that belong to the same ADAS-Cog items.

3.3. Measurement invariance of the ADAS-Cog items

Differential item functioning (DIF) was performed to investigate measurement invariance of the ADAS-Cog item characteristic functions due to gender (men/women), years of education (less/greater than 13 years), APOE-$\epsilon 4$ genotype (presence/absence of an $\epsilon 4$ allele), and the status of concomitant AChEI therapy (yes/no). All patients undergoing any of the AChEI medications (donepezil, rivastigmine, and galantamine) were labeled as positive for concomitant AChEI therapy. For every DIF factor, ADAS-Cog item characteristic functions were estimated separately inside each patient group and parameter estimates $\mathbf{\Psi}$ were compared using the Lord's Wald chi-square test with false discovery rate correction [3]. Before comparison, parameter estimates of patients groups were linearly transformed to a common scale by equating the means

and variances of item difficulties across all the groups. If parameter estimates of certain ADAS-Cog items were found to be significantly different between patient groups, those items were flagged as potentially suffering from measurement variance. The ADAS-Cog items that did not show any significant differences in parameter estimates were anchored by constraining their estimates to be equal across the patient groups. After item anchoring, parameters were re-estimated for all the ADAS-Cog items flagged as potentially suffering from measurement variance to validate if significant differences in parameters still exist between the patient groups. For DIF analysis, the sample size was kept similar across patient groups by randomly selecting patients from bigger patient groups.

The sample variance of the ADAS-Cog item characteristic functions was estimated by conducting 1000 bootstrap replications of estimation of item parameters $\boldsymbol{\Psi}$ with sample replacement. The large sample of patients considered in this study with diverse demographic and clinical characteristics provides a good representation of the overall variability in mild-to-moderate Alzheimer's patient population. Therefore, bootstrapping provides a rough estimate on the expected variability in the ADAS-Cog item characteristic functions if different samples of Alzheimer's patients are considered for IRT model estimation. The ADAS-Cog item characteristic functions showed tight agreement across 1000 bootstrap replications of model estimation (figures S2, S3, and S4), which suggests that there is little sample variance across different patient samples considered for estimation.

4. Measurement of cognitive impairment in patients

4.1. ADAS-Cog scoring methodology based on IRT modeling (ADAS-CogIRT)

The ADAS-CogIRT scoring methodology uses the ADAS-Cog item characteristic functions to measure cognitive impairment in patients based on their ADAS-Cog item response patterns. Given a patient's responses to the ADAS-Cog items $\boldsymbol{x_i} = (x_{i1}, \ldots, x_{in})$, cognitive impairment is measured as the values of the latent traits $\boldsymbol{\theta_i} = (\theta_i^{memory}, \theta_i^{language}, \theta_i^{praxis})$ that have the maximum likelihood of observing the ADAS-Cog item responses $\boldsymbol{x_i} = (x_{i1}, \ldots, x_{in})$:

$$L(\boldsymbol{x_i}|\boldsymbol{\theta_i}) = \sum_{j=1}^{n} \log(P(x_{ij}|\boldsymbol{\theta_i}, \boldsymbol{\Psi}))$$

$$\hat{\boldsymbol{\theta}}_{\boldsymbol{i}} = \arg\max_{\boldsymbol{\theta}} L(\boldsymbol{\theta_i}) \tag{3}$$

where, $L(\boldsymbol{x_i}|\boldsymbol{\theta_i})$ denotes the log-likelihood of observing the ADAS-Cog item responses $\boldsymbol{x_i}$ in a patient with cognitive impairment $\boldsymbol{\theta_i}$. $\boldsymbol{\Psi}$ denotes the parameters of the ADAS-Cog item characteristic functions after adjusting for measurement bias of the ADAS-Cog items due to patient-level factors. The updated ADAS-Cog item characteristic functions with adjustments for patient-level factors were defined as:

$$P(x_{ij} = 1|\boldsymbol{\theta_i}, \boldsymbol{\alpha_j}, d_j, g_j) = g_j + \frac{(1 - g_j)}{1 + \exp[-(\boldsymbol{\alpha_j^T}\boldsymbol{\theta_i} + \boldsymbol{W_i^T}\boldsymbol{\tau_j^T}\boldsymbol{\theta_i} + d_j + \boldsymbol{Z_i}\boldsymbol{\delta_j})]} \tag{4}$$

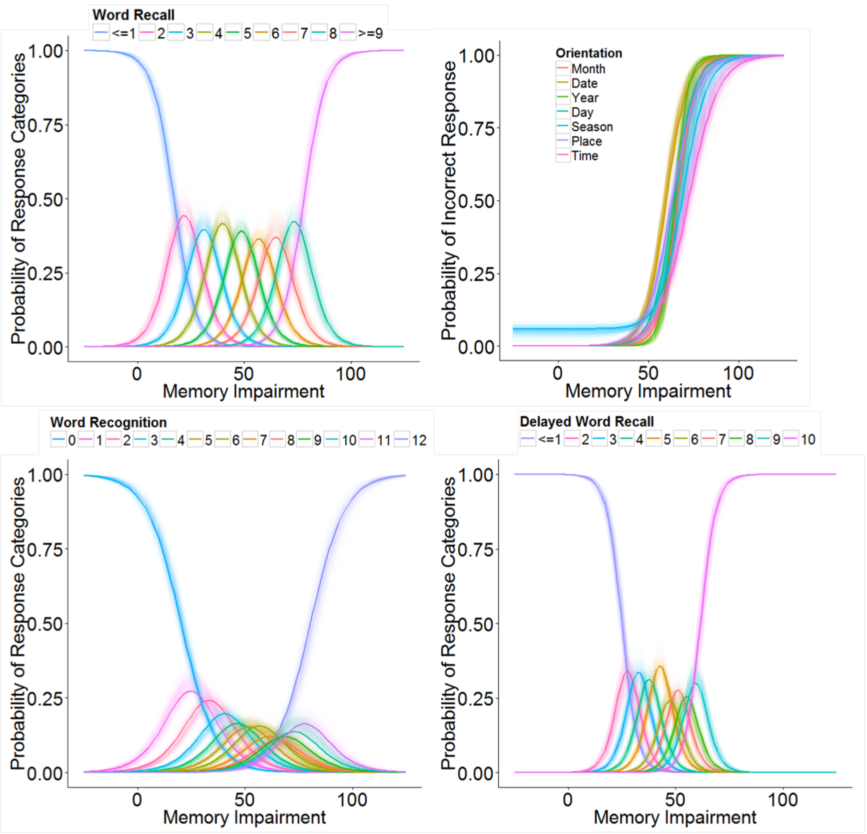

Figure S2: Item characteristic functions of memory items: Plots showing item characteristic functions (solid lines) of the ADAS-Cog items that measure memory impairment. The faint lines show variability in the item characteristic functions from 1000 bootstrap replications of parameter estimation with sample replacement.

where the fixed effects $\boldsymbol{\tau_j}$ and $\boldsymbol{\delta_j}$ denote adjustments in the ADAS-Cog item slopes and intercepts to account for measurement bias due to patient-level factors with $\boldsymbol{W_i}$ and $\boldsymbol{Z_i}$ as the associated design matrices, respectively.

The measurement scales for memory, language, and praxis impairment were defined such that the impairment scores in Alzheimer's disease patients have means of 50 points and standard deviations of 15 points, respectively. In item response theory, the parameters of the item characteristic functions are estimated based on the assumption that the latent traits (or person abilities) have means of 0 and standard deviations of 1. For defining the measurement scales, the parameters of the ADAS-Cog item characteristic functions were linearly scaled as follows:

$$a_{new} = \frac{a_{old}}{x}$$

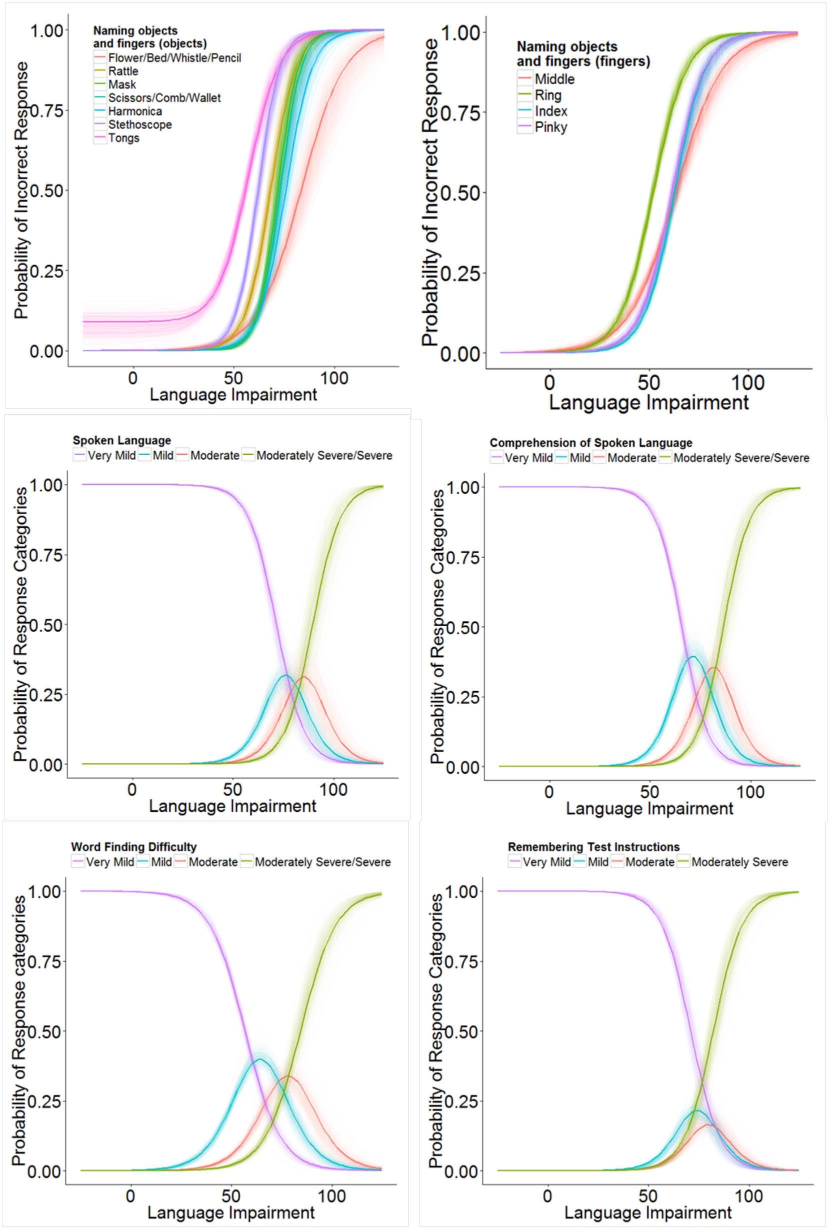

Figure S3: Item characteristic functions of language items: Plots showing item characteristic functions (solid lines) of the ADAS-Cog items that measure language impairment. The faint lines show variability in the item characteristic functions from 1000 bootstrap replications of parameter estimation with sample replacement.

$$d_{new} = \quad d_{old} - a_{old} \times \frac{y}{x} \tag{5}$$

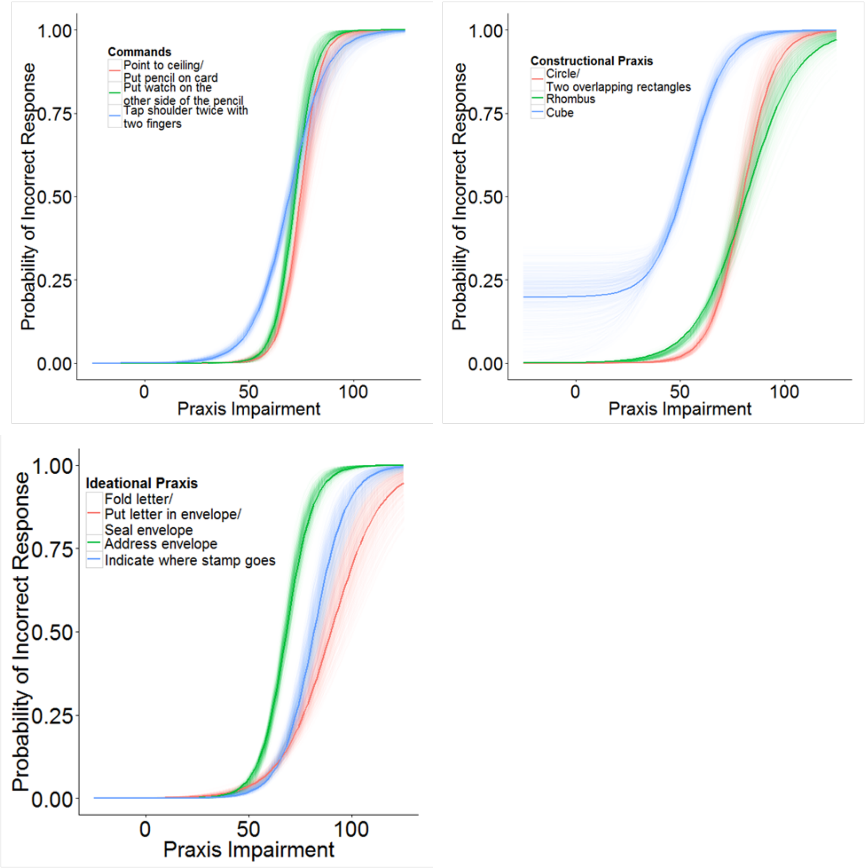

Figure S4: Item characteristic functions of praxis items: Plots showing item characteristic functions (solid lines) of the ADAS-Cog items that measure praxis impairment. The faint lines show variability in the item characteristic functions from 1000 bootstrap replications of parameter estimation with sample replacement.

where, a_{old} and d_{old} denotes the item slopes and item intercepts as estimated by item response theory based on the assumption of $\theta \sim N(0, 1)$. The parameters of the ADAS-Cog item characteristic functions were scaled (using $x = 15$ and $y = 50$ in equations (5)) such that the estimated memory, language, and praxis impairment θ_i^{new} have means of 50 points and standard deviations of 15 points.

4.2. Accuracy of the ADAS-CogIRT methodology for measuring cognitive impairment

The ADAS-CogIRT methodology in (3) was used to separately estimate cognitive impairment in patients at the baseline, 6 months, and 12 months visits using their ADAS-Cog responses. Assuming linear progression over time, cognitive impairment at the 24-months visit was estimated for every patient by fitting a linear regression line to the estimates of cognitive impairment from the

earlier visits. The estimated cognitive impairment at the 24-months visit was used to predict the ADAS-Cog item responses of patients as:

$$\hat{x_{ij}} = E\left[x_{ij}\right] = \sum_{k=0}^{C_j-1} k \times P(x_{ij} = k|\hat{\boldsymbol{\theta_i}}, \boldsymbol{\Psi}) \tag{6}$$

where $k = \{0, \ldots, C_j - 1\}$ are the response categories of the ADAS-Cog item j and $\hat{\boldsymbol{\theta_i}}$ represents the estimated cognitive impairment in patient i at the 24-months visit. $P(x_{ij} = k|\hat{\boldsymbol{\theta_i}}, \boldsymbol{\Psi})$ was calculated for every patient using the ADAS-Cog item characteristic functions in equation (2).

The current scoring methodology measures cognitive impairment in patients by adding scores across the ADAS-Cog items. Therefore, using the current scoring methodology, the total ADAS-Cog scores at the 24-months visit can be predicted by simply fitting a linear regression line to the total ADAS-Cog scores of patients from the earlier visits. The prediction accuracy of the ADAS-CogIRT methodology was assessed by calculating the root mean squared error (RMSE) between the observed total ADAS-Cog scores $\sum_j x_{ij}$ and the predicted total ADAS-Cog scores $\sum_j \hat{x_{ij}}$:

$$RMSE_{ADAS} = \sqrt{\frac{\sum_i (\sum_j x_{ij} - \sum_j \hat{x_{ij}})^2}{N_T}} \tag{7}$$

where N_T represents the number of patients belonging to the treatment arms of the 5 ADCS clinical trials.

4.3. Precision of the ADAS-CogIRT methodology for measuring cognitive impairment

The precision of the ADAS-CogIRT methodology was evaluated by calculating the item information functions of the ADAS-Cog items:

$$I_j(\theta) = \sum_{k=0}^{C_j-1} \frac{1}{P_{jk}(\theta)} \left(\frac{dP_{jk}(\theta)}{d\theta}\right)^2 \tag{8}$$

where $P_{jk}(\theta)$ represents the probability of kth response by a patient with cognitive impairment θ to the jth ADAS-Cog item, as defined in equation (2). The cumulative Fisher information across all the ADAS-Cog items was used to estimate the expected standard error associated with the measurement of different levels of cognitive impairment.

5. Improving the sensitivity of the ADAS-Cog in clinical trials

5.1. Application of the ADAS-CogIRT methodology in clinical trials

We propose a generalized mixed-effects approach for using the ADAS-CogIRT methodology in clinical trials. Besides estimating the baseline cognitive impairment, this approach also estimates the rates of progression of cognitive impairment in patients using their longitudinal ADAS-Cog responses. In longitudinal

settings, the ADAS-Cog item characteristic functions are represented as

$$P(x_{ij}^t = 1|\boldsymbol{\theta_i^t}, \boldsymbol{\alpha_j}, d_j, g_j) = g_j + \frac{(1-g_j)}{1+\exp[-(\boldsymbol{\alpha_j^T}\boldsymbol{\theta_i^t} + \boldsymbol{W_i^T}\boldsymbol{\tau_j^T}\boldsymbol{\theta_i^t} + d_j + \boldsymbol{Z_i}\boldsymbol{\delta_j})]} \quad (9)$$

where x_{ij}^t and $\boldsymbol{\theta_i^t}$ represent the ADAS-Cog item responses and cognitive impairment of patients at time t. For clinical trials, cognitive impairment in patients are assumed to progress linearly with time:

$$\boldsymbol{\theta_i^t} = \boldsymbol{\theta_i^0} + \boldsymbol{r_i} \times t \quad (10)$$

where $\boldsymbol{\theta_i^0}$ and $\boldsymbol{r_i}$ represent baseline cognitive impairment and progression rates in patients. Baseline cognitive impairment $\boldsymbol{\theta_i^0}$ and progression rates $\boldsymbol{r_i}$ are modeled as mixed effects:

$$\begin{aligned} \boldsymbol{\theta_i^0} &= \boldsymbol{\mu_\theta} + \boldsymbol{\beta_{Arm}} \times (\text{Arm}_i) + \boldsymbol{\beta_{Patient}} \times (P_i) + \boldsymbol{\varepsilon_{i,\theta}} \\ \boldsymbol{r_i} &= \boldsymbol{\mu_r} + \boldsymbol{\gamma_{Arm}} \times (\text{Arm}_i) + \boldsymbol{\gamma_{Patient}} \times (P_i) + \boldsymbol{\varepsilon_{i,r}} \\ &\begin{pmatrix} \varepsilon_{i,\theta} \\ \varepsilon_{i,r} \end{pmatrix} \sim N\big(0, \begin{bmatrix} \Sigma_{\theta,\theta} & \Sigma_{\theta,r} \\ \Sigma_{\theta,r} & \Sigma_{r,r} \end{bmatrix}\big) \end{aligned} \quad (11)$$

where, $\boldsymbol{\mu_\theta}$ and $\boldsymbol{\mu_r}$ represent the average levels of baseline cognitive impairment and progression rates across patients in the placebo arm. The trial arm information of patients is included in the form of a categorical covariate Arm_i such that $\text{Arm}_i = \begin{cases} 0 & \text{if placebo arm} \\ 1 & \text{if treatment arm} \end{cases}$. The fixed effects $\boldsymbol{\beta_{Arm}}$ and $\boldsymbol{\gamma_{Arm}}$ measure differences in the average levels of baseline cognitive impairment and progression rates of patients between the placebo and treatment arms. Patient-level covariates P_i are included to model systematic variability in baseline cognitive impairment and progression rates with $\boldsymbol{\beta_{Patient}}$ and $\boldsymbol{\gamma_{Patient}}$ representing the associated fixed effects. Random effects $\boldsymbol{\varepsilon_{i,\theta}}$ and $\boldsymbol{\varepsilon_{i,r}}$ are included to model random variations in baseline cognitive impairment and progression rates across patients. The cognitive impairment and progression rates in Alzheimer's patients are inter-correlated and, therefore, the random effects $\boldsymbol{\varepsilon_{i,\theta}}$ and $\boldsymbol{\varepsilon_{i,r}}$ are allowed to covary. The parameters of the proposed methodology are estimated using maximum likelihood estimation with adaptive Gauss-Hermite quadrature.

5.2. Design of clinical trial simulations

The parameters for simulating clinical trials in this study were obtained by analyzing the ADAS-Cog responses of mild-to-moderate Alzheimer's patients (total ADAS-Cog scores of 25±10) in the placebo arms of ADCS and CAMD trials using a generalized mixed-effects approach similar to (11). Besides the patient-level random effects, nested study-level random effects were also included to model variability in disease stages, where these clinical trials were focused. The parameters estimated for simulating clinical trials were average baseline cognitive impairment and progression rates ($\boldsymbol{\mu_\theta}$, $\boldsymbol{\mu_r}$), random inter-patient variability in baseline cognitive impairment and progression rates ($\Sigma_{\theta,\theta}$,

$\Sigma_{r,r}$, $\Sigma_{\theta,r}$), and systematic variability in baseline cognitive impairment and progression rates due to patient-factors ($\beta_{Patient}$, $\gamma_{Patient}$).

The average baseline memory, language, and praxis impairment in mild-to-moderate Alzheimer's patients were estimated as 56.50, 57.83, and 60.27 points. The random inter-patient variability (standard deviation) in baseline memory, language, and praxis impairment were estimated to be 6.31, 7.91, and 8.47 points, respectively. The annual rates of progression in memory, language, and praxis impairment had averages of 2.61, 3.03, and 2.10 points and inter-patient variability of 3.83, 5.56, and 5.04 points, respectively. Patient age was associated with more pronounced baseline cognitive impairment ($\beta_{Age,Mem} = 0.19$, $\beta_{Age,Lang} = 0.10$); however, the progression rates decreased with patient age ($\gamma_{Age,Mem} = -0.08$, $\gamma_{Age,Lang} = -0.09$, $\gamma_{Age,Prax} = -0.16$). APOE-$\epsilon$4 genotype was associated with higher baseline memory impairment ($\beta_{APOE,Mem} = 2.83$); however, the progression rates of impairment in all the cognitive domains increased with the presence of an ϵ4 allele ($\gamma_{APOE,Mem} = 0.97$, $\gamma_{APOE,Lang} = 1.92$, $\gamma_{APOE,Prax} = 1.17$). In the Cox proportional hazards model for patient dropout, the progression rates in various cognitive domains were found to increase the hazard by factors of 2.77 (memory), 1.42 (language), and 2.92 (praxis), while age increased the dropout hazard by a factor of 1.02.

A large sample of 10000 patients was simulated with normally distributed levels of baseline cognitive impairment and progression rates (using $\boldsymbol{\mu_\theta}$, $\boldsymbol{\mu_r}$, $\Sigma_{\theta,\theta}$, $\Sigma_{r,r}$ and $\Sigma_{\theta,r}$) to represent the population of mild-to-moderate Alzheimer's patients. Based on the sample characteristics of previous trials, 58.5% patients were randomly labeled as APOE-ϵ4 positive, 52.8% were randomly labeled as women, and patient ages were simulated as normally distributed with mean of 74.7 years and standard deviation of 8.54 years. For each clinical trial simulation, S patients were selected at random from this population and randomly distributed between the placebo and treatment arms. A hypothetical treatment effect of effect size d (in terms of Cohen's $d = \gamma_{Arm}/\sqrt{\Sigma_{r,r}}$) was introduced in the progression rates of impairment in randomly chosen subset of cognitive domains of patients belonging to the treatment arm. The dropout of patients from clinical trials was simulated by using the estimated Cox proportional hazards model. The baseline cognitive impairment and progression rates of patients were used to calculate their longitudinal levels of cognitive impairment at each visit until the duration of the trial. The longitudinal ADAS-Cog responses of patients were simulated using the ADAS-Cog item characteristic functions. The parameters of the ADAS-Cog item characteristic functions were randomly perturbed using the estimated standard errors of the parameters in order to reduce the extent of specification bias from using the same parameters for simulating and analyzing the data. For example, if the slope of an item is estimated as μ_α with a standard error of σ_α, the item parameter was perturbed for every trial simulation by randomly sampling a value from the normal distribution $\alpha \sim N(\mu_\alpha, \sigma_\alpha^2)$.

The ADAS-Cog response data from every simulation of clinical trial was modeled using the proposed generalized mixed-effects approach in equation (11). The statistical significance of the treatment effect in a clinical trial was assessed

using z-statistic with correction for multiple comparisons due to multiple cognitive domains being assessed.

5.3. Sensitivity analysis using clinical trial simulations

In the paper, we have evaluated the statistical power of the proposed ADAS-CogIRT and the ANCOVA methodologies for detecting treatment effects in clinical trials using simulation experiments. We additionally evaluated a single latent trait variant of the ADAS-CogIRT in clinical trial simulations to illustrate the significance of separately measuring impairment in the memory, language, and praxis cognitive domains. Similar to the results reported in [4], we observed that a single latent trait IRT model has better statistical power than the currently used ANCOVA methodology. However, the proposed ADAS-CogIRT methodology illustrated significantly better statistical power than the single latent trait model (figures S5 and S6). The lower power of the single latent trait model is primarily because the single trait measures a weighted average of impairment across the three cognitive domains. As a result, in clinical trials with non-uniform treatment effects across the cognitive domains, the single latent trait method obscures the detection of treatment effects.

5.4. Sensitivity analysis using a real clinical trial

The level of treatment effects detected by the ADAS-CogIRT and the ANCOVA methodologies were estimated as $d = \gamma_{Arm}/\sqrt{\Sigma_{r,r}}$.

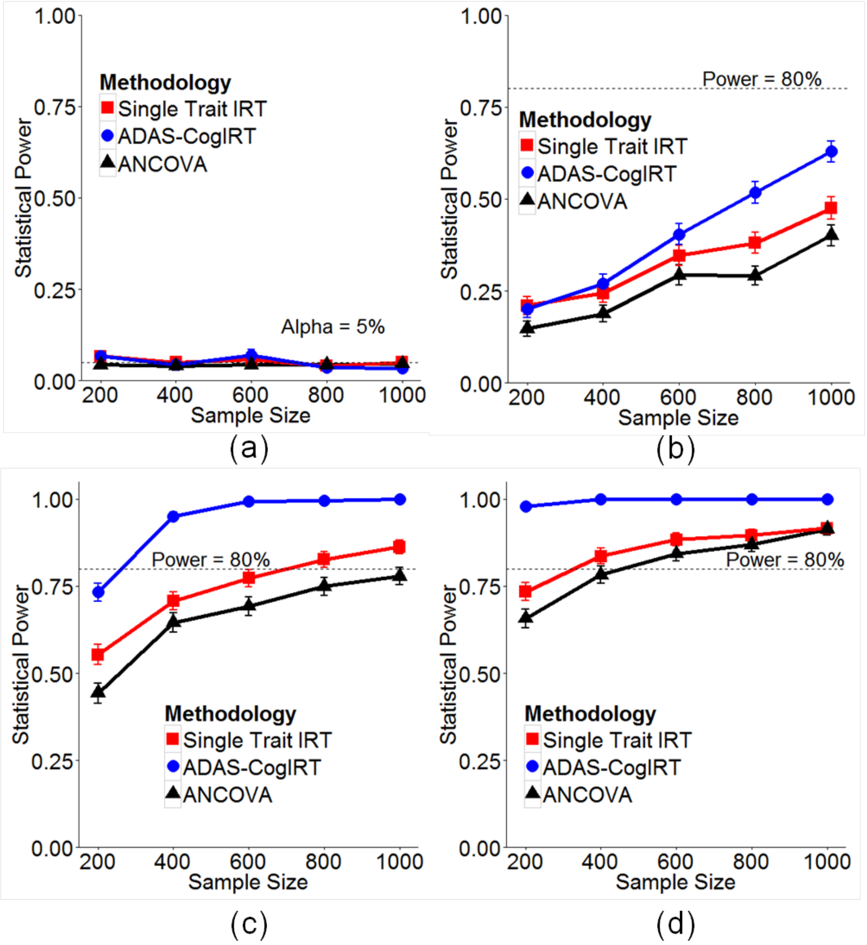

Figure S5: Statistical power against sample size: Plots showing the relationship between the statistical power of the ADAS-CogIRT, single latent trait variant of the ADAS-CogIRT and ANCOVA methodologies and sample size for hypothetical treatment levels of (a) $d = 0$, (b) $d = 0.2$, (c) $d = 0.5$, and (d) $d = 0.8$. The trial duration was fixed at 24 months.

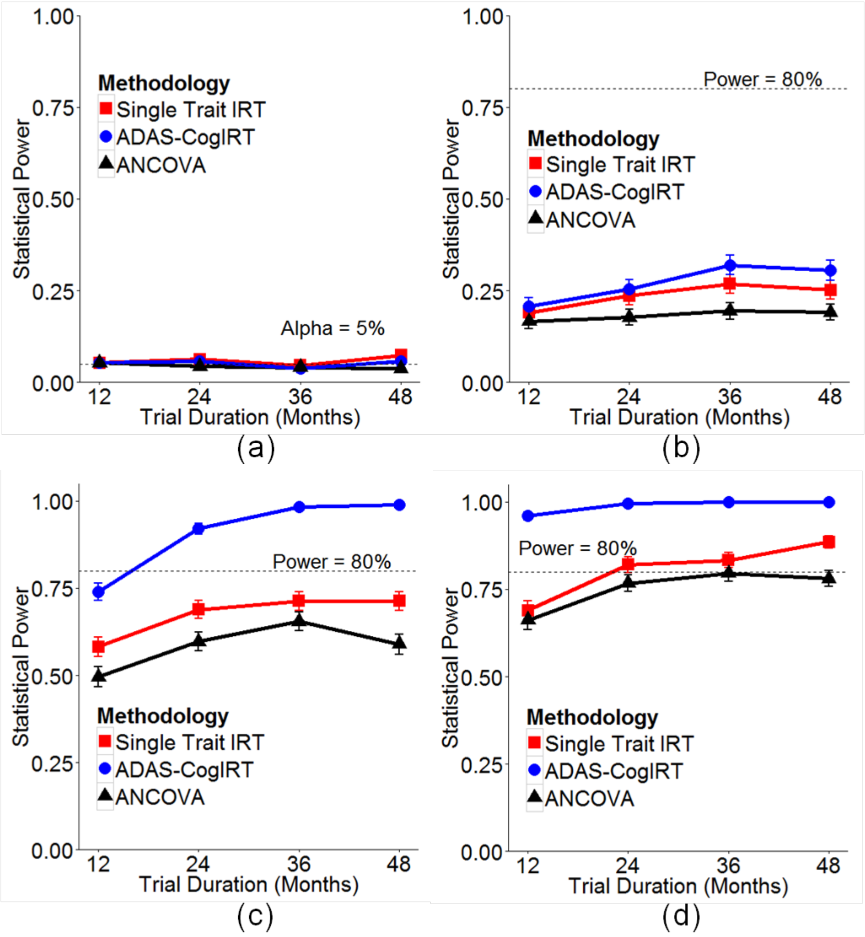

Figure S6: Statistical power against trial duration: Plots showing the relationship between the statistical power of the ADAS-CogIRT, single latent trait variant of the ADAS-CogIRT and ANCOVA methodologies and duration of clinical trials for hypothetical treatment levels of (a) $d = 0$, (b) $d = 0.2$, (c) $d = 0.5$, and (d) $d = 0.8$. The sample size was fixed at 400 patients.

References

[1] L. Cai, "Metropolis-Hastings Robbins-Monro algorithm for confirmatory item factor analysis," *Journal of Educational and Behavioral Statistics*, vol. 35, no. 3, pp. 307–335, 2010.

[2] R. D. Bock and M. Aitkin, "Marginal maximum likelihood estimation of item parameters: Application of an EM algorithm," *Psychometrika*, vol. 46, no. 4, pp. 443–459, 1981.

[3] F. M. Lord, *Applications of item response theory to practical testing problems.* Routledge, 1980.

[4] S. Ueckert, E. L. Plan, K. Ito, M. O. Karlsson, B. Corrigan, A. C. Hooker, A. D. N. Initiative, *et al.*, "Improved utilization of ADAS-cog assessment data through Item Response Theory based pharmacometric modeling," *Pharmaceutical Research*, vol. 31, no. 8, pp. 2152–2165, 2014.
